# Supplementary material for: A platform-independent framework for phenotyping of multiplex tissue imaging data
Source: PLoS Comput Biol. 2023 Sep 21;19(9):e1011432. doi: 10.1371/journal.pcbi.1011432 (PMC10547204; doi:10.1371/journal.pcbi.1011432)
Supplement: S1 Text — Fig A. Top: Marker expression per cell measured using the images (top row) and the FR maps (middle row) overlaid on the tSNE plot for rest of the immune and tumor markers. The bottom row demonstrate correlation between marker expression per cell from raw images (x-axis) and FR maps (y-axis). The strength and direction of these correlations are quantified using Spearman’s rank correlation coefficient. Left: Cells are sorted by cell types identified by our clustering (y-axis) against marker expression (x-axis). Expression values for each marker are measured from the FR maps. Stacked bar plot shows the abundance of each cell type in the dataset. Right: Cell-cell comparison between the cell type identified by the TNBC study versus our framework (left panel). Numbers in table cells indicate the percentage of cells in the dataset where columns and rows, respectively, compare their identified types by the baseline and our framework. Fig B. Quantifying the correlation between the raw image pixel intensity and FR maps for marker-positive cells. Correlation plots illustrating marker expression per cell from raw images (x-axis) against FR maps (y-axis) are presented in rows 1 and 3. Specifically, these plots focus on cells where the average FR map values per cell exceed a selected threshold value. To rationalize the chosen threshold values (indicated by red lines), histograms displaying average values per cell measured from FR maps are included, delineating positive cells for a given marker from the negative ones. We note that the mapping from pixel values in the raw image to the FR map is influenced not only by pixel intensity but also by the spatial information of surrounding pixels. Consequently, positive signals may yield large values in the FR map; however, as these values increase in the raw image, the values level off in the FR map. This characteristic does not present any issues, as our framework is not designed to assess the level of expression for functional markers, [file pcbi.1011432.s001.pdf]

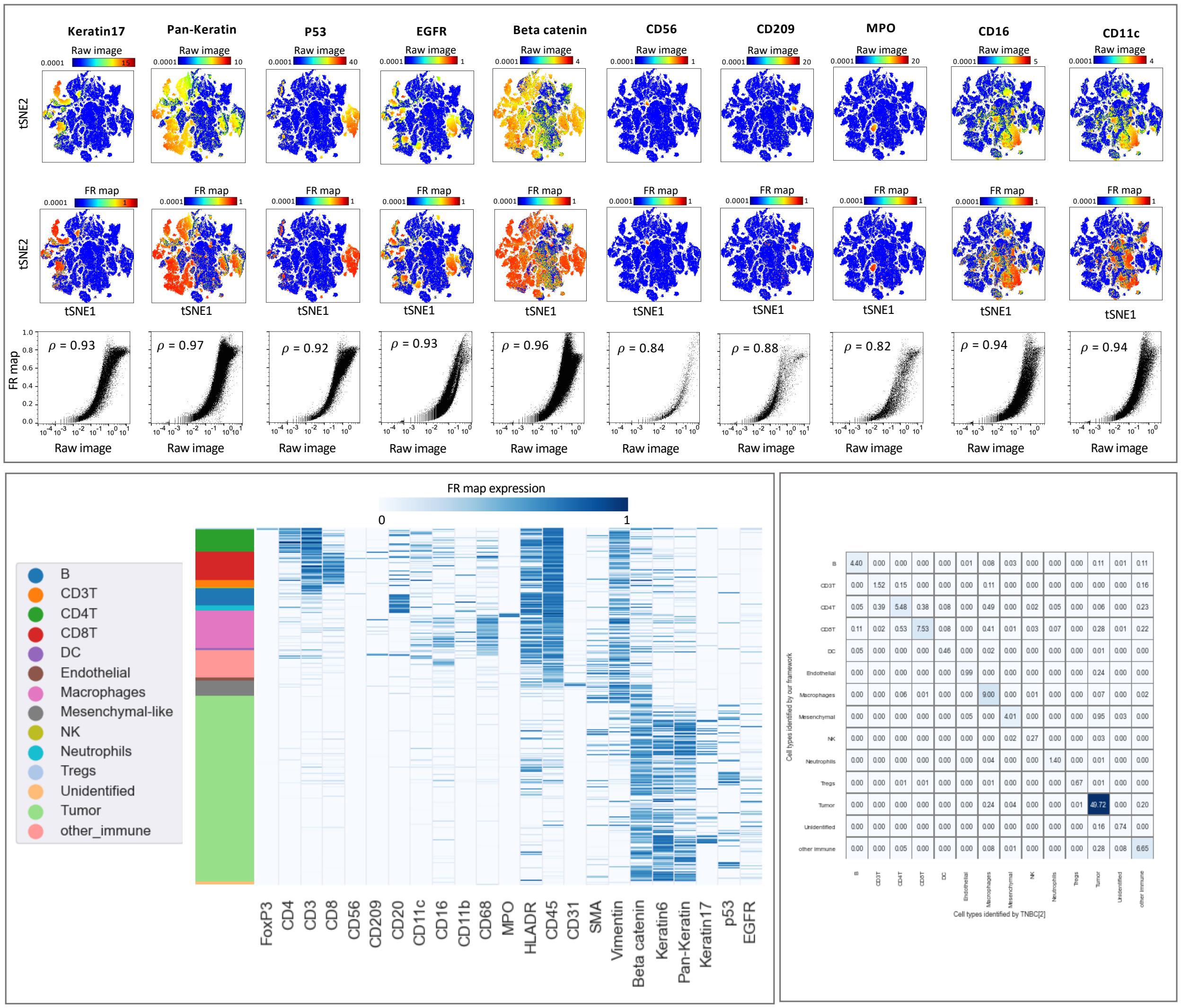

Fig A. Top: Marker expression per cell measured using the images (top row) and the FR maps (middle row) overlaid on the tSNE plot for rest of the immune and tumor markers. The bottom row demonstrate correlation between marker expression per cell from raw images (x-axis) and FR maps (y-axis). The strength and direction of these correlations are quantified using Spearman's rank correlation coefficient. Left: Cells are sorted by cell types identified by our clustering (y-axis) against marker expression (x-axis). Expression values for each marker are measured from the FR maps. Stacked bar plot shows the abundance of each cell type in the dataset. Right: Cell-cell comparison between the cell type identified by the TNBC study versus our framework (left panel). Numbers in table cells indicate the percentage of cells in the dataset where columns and rows, respectively, compare their identified types by the baseline and our framework.

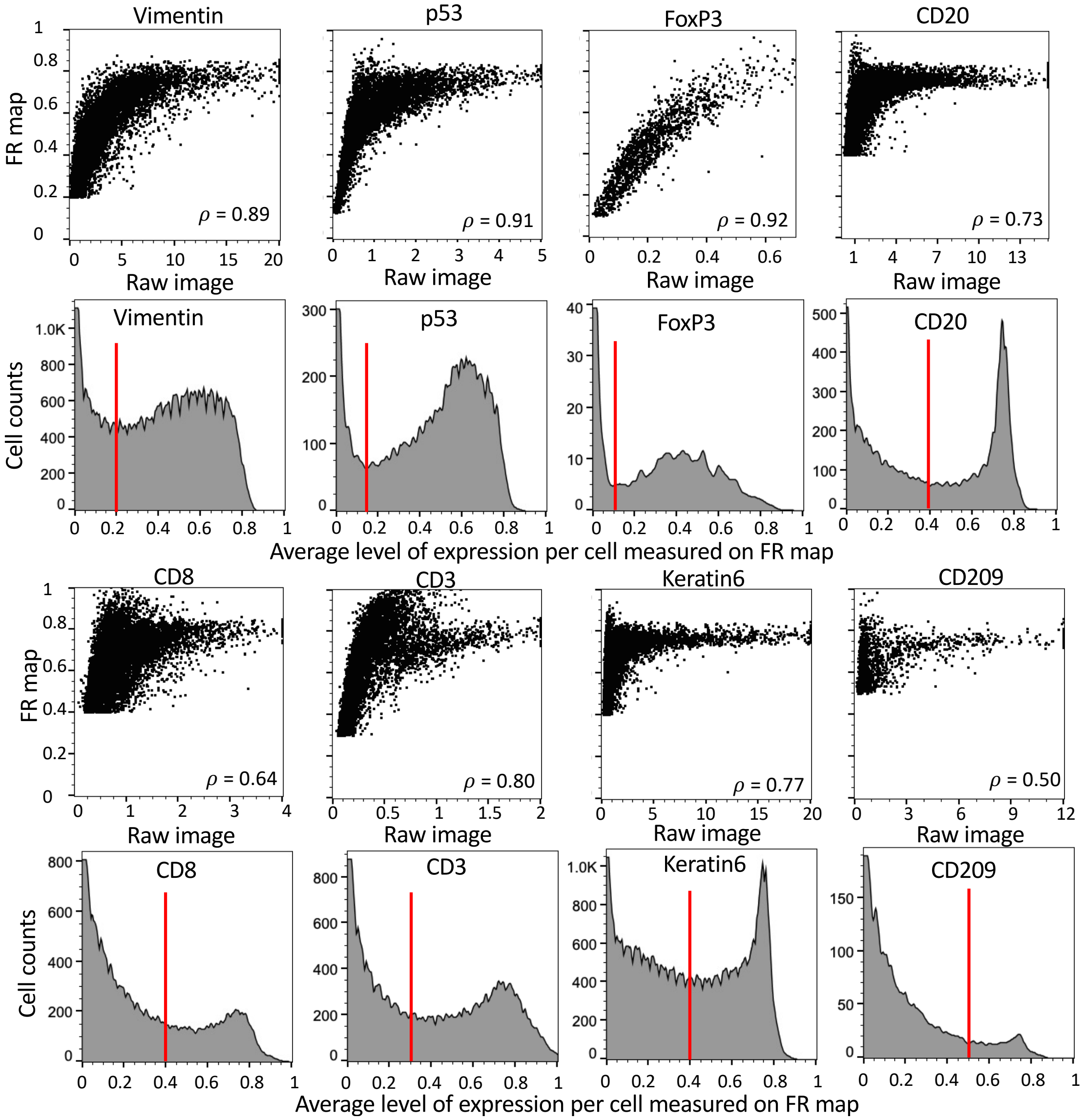

Fig B. Quantifying the correlation between the raw image pixel intensity and FR maps for marker-positive cells. Correlation plots illustrating marker expression per cell from raw images (x-axis) against FR maps (y-axis) are presented in rows 1 and 3. Specifically, these plots focus on cells where the average FR map values per cell exceed a selected threshold value. To rationalize the chosen threshold values (indicated by red lines), histograms displaying average values per cell measured from FR maps are included, delineating positive cells for a given marker from the negative ones. We note that the mapping from pixel values in the raw image to the FR map is influenced not only by pixel intensity but also by the spatial information of surrounding pixels. Consequently, positive signals may yield large values in the FR map; however, as these values increase in the raw image, the values level off in the FR map. This characteristic does not present any issues, as our framework is not designed to assess the level of expression for functional markers, but rather to determine whether a cell is positive or negative for a given marker.

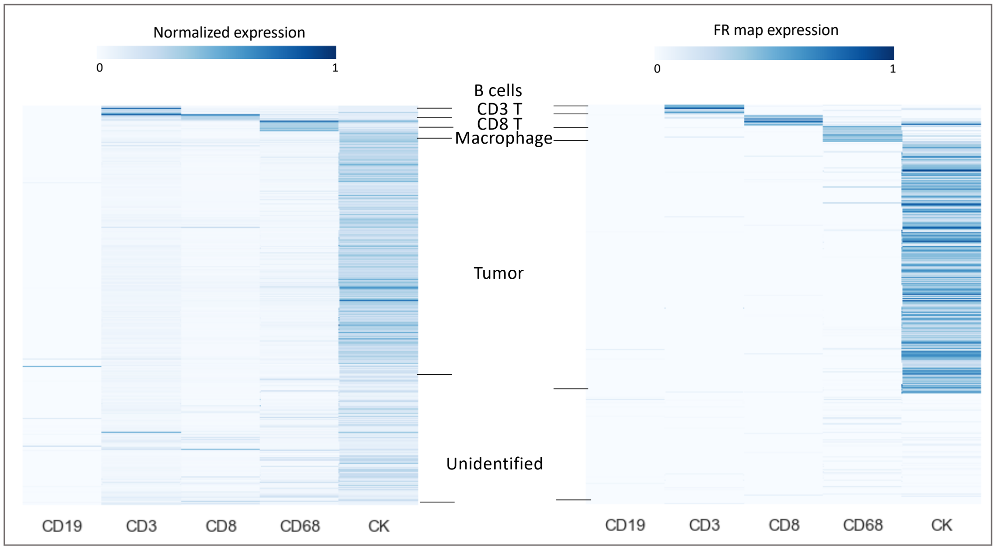

Fig C. Cell types clustered by marker expression. Expression values for each marker are measured from the raw image (left) and the FR maps (right).

Table A. Classification features extracted for the breast cancer and the ovarian cancer datasets.

Feature	Number of extracted features	Notes
Gaussian blur	5 (σ = 1, 2, 4, 8, 16)	Performs 5 convolutions of Gaussian kernels with the 5 variations of σ and generates 5 features for each image.
Sobel filter	5	Calculates an approximate of the gradient of the image intensity at each pixel for each image after applying 5 variations of Gaussian blurs; generates 5 features for each image.
Difference of Gaussians	10	Calculates two Gaussian blur images from the original image and subtracts one from the other. Thus, with 5 variations of Gaussian blur, 10 features are generated for each image.
Mean	5	Mean is calculated from the pixels within a radius of σ pixels from the target pixel and the target pixel is set to that mean value. With 5 variations of Gaussian blur, 5 features are generated for each image.
Median	5	Median is calculated from the pixels within a radius of σ pixels from the target pixel and the target pixel is set to that median value. With 5 variations of Gaussian blur, 5 features are generated for each image.
Entropy	20	Within radius σ around each pixel, generates the histogram of that circle using n = 32, 64, 128, 256 as the number of bins and calculates the entropy as $\sum -p*\log2(p)$, where p is the probability of each collection in the histogram. With 5 variations of Gaussian blurs adds 20 features for each image.

Table B. Classification features from Table A in descending order of importance for CD20 marker from breast cancer dataset.

Features	Information gain ratio score
Original image	0.3192
Gaussian_blur_2.0	0.8789
Gaussian_blur_4.0	0.8744
Entropy_2_64	0.866
Entropy_2_256	0.8628
Entropy_2_128	0.8625
Difference_of_gaussians_2.0_1.0	0.8612
Mean_2.0	0.8607
Gaussian_blur_1.0	0.8547
Difference_of_gaussians_4.0_1.0	0.8373
Mean_4.0	0.8356
Entropy_1_256	0.8328
Entropy_1_128	0.8328
Mean_1.0	0.8294
Entropy_1_64	0.8251
Entropy_4_256	0.8251
Entropy_4_128	0.8248
Sobel_filter_1.0	0.8242
Entropy_4_64	0.8201
Gaussian_blur_8.0	0.7958
Difference_of_gaussians_16.0_8.0	0.7953
Sobel_filter_2.0	0.7788
Difference_of_gaussians_8.0_1.0	0.7539
Difference_of_gaussians_16.0_4.0	0.7483
Difference_of_gaussians_4.0_2.0	0.7308
Sobel_filter_0.0	0.7235
Difference_of_gaussians_8.0_4.0	0.7045
Mean_8.0	0.7001
Entropy_8_256	0.6931
Entropy_8_128	0.6928
Entropy_8_64	0.6894
Difference_of_gaussians_16.0_2.0	0.6782
Sobel_filter_4.0	0.6776
Difference_of_gaussians_8.0_2.0	0.6709
Entropy_2_32	0.6708
Gaussian_blur_16.0	0.6663
Entropy_4_32	0.6334
Difference_of_gaussians_16.0_1.0	0.6231
Entropy_1_32	0.5938
Entropy_16_128	0.5761
Entropy_16_64	0.5726
Entropy_16_256	0.5704
Sobel_filter_8.0	0.5548
Mean_16.0	0.5532
Entropy_8_32	0.5281
Entropy_16_32	0.4512
Sobel_filter_16.0	0.3255
Median_1.0	0.3148
Median_2.0	0.295
Median_4.0	0.1997
Median_8.0	0.0309
Median_16.0	0

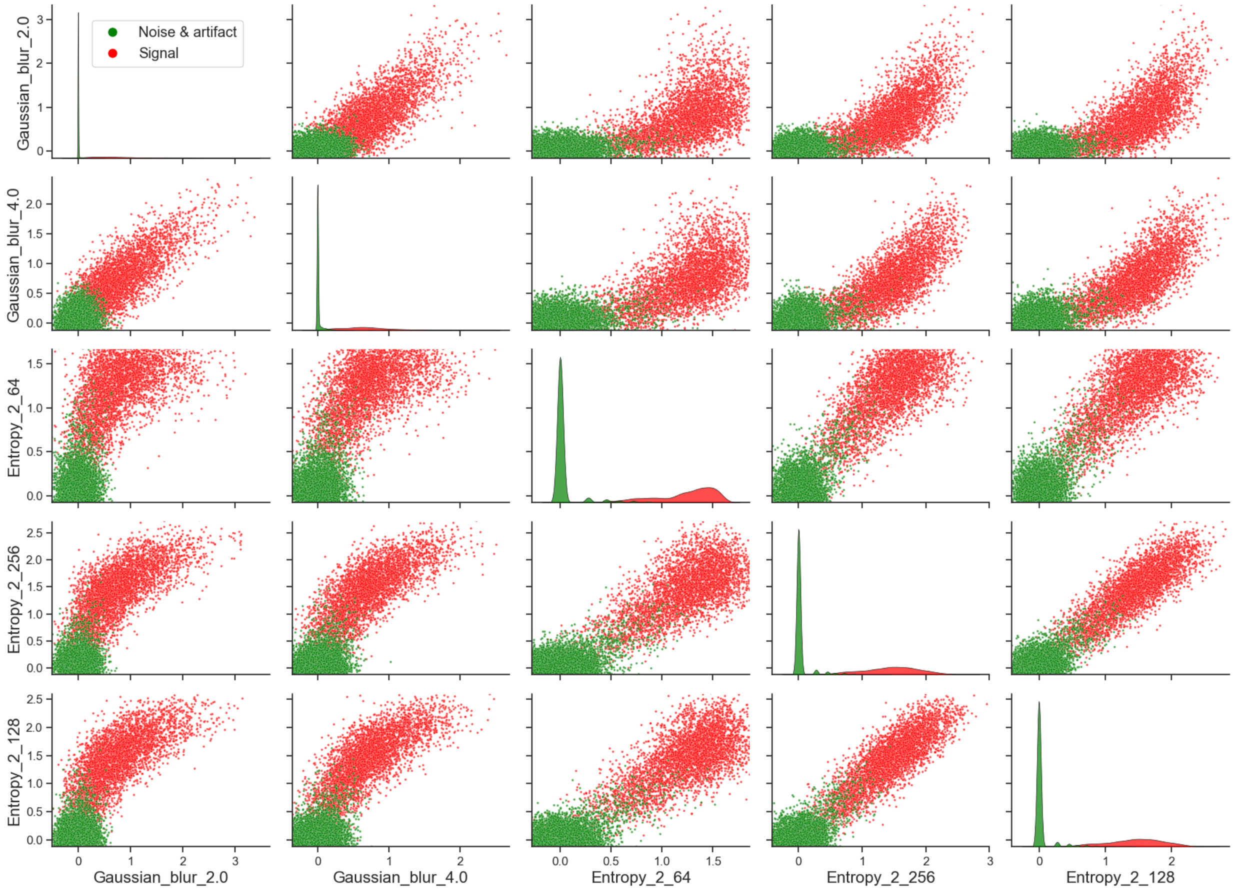

Fig D. The figure displays the top 5 features utilized for pixel classification of images from the CD20 channel from the breast cancer dataset.
