# Supplementary material for: A platform-independent framework for phenotyping of multiplex tissue imaging data
Source: PLoS Comput Biol. 2023 Sep 21;19(9):e1011432. doi: 10.1371/journal.pcbi.1011432 (PMC10547204; doi:10.1371/journal.pcbi.1011432)
Supplement: Attachment — Submitted filename: Response_Letter_PCOMPBIOL_D_22_01763 (1).pdf [file pcbi.1011432.s003.pdf]

Responses to the Reviewers' Comments on PLOS Computational Biology with reference number PCOMPBIOL-D-22-01763

"A Platform-Independent Framework for Phenotyping of Multiplex Tissue Imaging Data"

By Mansooreh Ahmadian, Christian Rickert, Angela Minic, Julia Wrobel, Benjamin G Bitler, Fuyong Xing, Michael Angelo, Elena W.Y Hsieh, Debashis Ghosh, Kimberly R Jordan

We would like to thank the editor and the three reviewers for their constructive comments, which have been helpful in the revision and improvements to the quality and technical contents of this paper. We have carefully considered and addressed all the comments and suggestions from the reviewers, as outlined in our response below. Reviewers' comments are indicated in gray italics, followed by our corresponding responses. Please note that, unless stated otherwise, all page numbers mentioned refer to the revised manuscript. In this response letter, Figures, Tables, and References are referred to as "Figure F1," "Table T1," and "Reference R1," respectively. To avoid confusion, references to figures and tables in the manuscript are made using the usual format (e.g., Figure 2, Table 3). Additionally, in the revised manuscript, all revisions are indicated using blue font for easy identification.

Editor's comment

*Thank you very much for submitting your manuscript "A Platform-Independent Framework for Phenotyping of Multiplex Tissue Imaging Data" for consideration at PLOS Computational Biology. As with all papers reviewed by the journal, your manuscript was reviewed by members of the editorial board and by several independent reviewers. In light of the reviews (below this email), we would like to invite the resubmission of a significantly-revised version that takes into account the reviewers' comments. In addition to addressing all reviewers' comments, please ensure that details on training, *along with training data* are made publicly available either on the project's GitHub repository or on another data sharing platform of your choice (eg Zenodo)*

before submitting the revised version. We cannot make any decision about publication until we have seen the revised manuscript and your response to the reviewers' comments. Your revised manuscript is also likely to be sent to reviewers for further evaluation. When you are ready to resubmit, please upload the following: [1] A letter containing a detailed list of your responses to the review comments and a description of the changes you have made in the manuscript. Please note while forming your response, if your article is accepted, you may have the opportunity to make the peer review history publicly available. The record will include editor decision letters (with reviews) and your responses to reviewer comments. If eligible, we will contact you to opt in or out. [2] Two versions of the revised manuscript: one with either highlights or tracked changes denoting where the text has been changed; the other a clean version (uploaded as the manuscript file).

Response to the Editor

We would like to thank the editor for handling the review process of our manuscript. We have thoroughly addressed each of the reviewers' comments and incorporated their valuable suggestions. As a result, the manuscript has undergone substantial revisions, leading to significant improvements. We would like to provide you with a summary of the major changes made in the revised manuscript. For a more comprehensive understanding, we kindly request that you refer to our response to the reviewers.

- We have extensively enhanced the description of the training and evaluation processes for our models. This includes providing detailed information on the generation of training sets, feature selection techniques, evaluation of feature contributions, cross-validation methods, metrics employed, as well as the computational complexity involved.
- To ensure transparency and reproducibility, we have made all data associated with the study readily available. This includes raw and processed data such as raw imaging data, feature representation maps, segmentation maps, and single-cell information tables. These data can be accessed in the GitHub repository dedicated to this manuscript.
- Quantitative measures have been added to the qualitative illustrations to provide a quantitative and statistical comparison between the baselines and our proposed approach.
- Several figures have undergone significant revisions, and additional figures and tables have been incorporated to ensure the inclusion of all pertinent details.

Response to Reviewer 1

The authors touch upon an interesting and timely topic which deals with different aspects of preprocessing of raw data from multiplexed imaging. They argue that downstream analysis of imaging data relies on artefact removal, denoising and normalization which could all affect cell type identification and sample-to-sample quantification. The propose a single-step cross platform solution which relies on pixel classification which includes denoising, artefact removal and normalization and does not rely on any user defined thresholds. In this method each pixel from each channel is classified as either signal or noise and this is outputted as probability which ranges from 0 to 1 (FR map) and the authors consider these values as normalized and use them for downstream analysis. While the manuscript is compact, easy to read and interesting there are some aspects that are unclear to me and need clarification.

Major comments#1- *It is unclear how the training and the validation of the approach were done. Did the authors train on the entire stack of the individual images? If so, then no validation of the approach is provided. Meaning, how does the classifier perform if it is provided data from a sample/patient it has never seen? Usually, the specificity and sensitivity of random forests drops then. The authors should make this clear and if the whole stack was used for training then the authors should rerun the classifier with a hold out test set which they can then use for testing. This has implications for the applicability of the approach as it would likely not be feasible to label 40 channels of projects that contain e.g. 400 images… Thus, knowing the performance on new data is crucial.*

We appreciate the opportunity to provide further details on our training and validation procedure and describe the general pipeline for generating FR maps and how we implemented it for this manuscript in this response.

First, we generate representative training stacks per channel using quantitative scores obtained from the CU-IScore scoring method. CU-IScore [R1] is a scoring system developed in-house and publicly available, based on the IHC-Profiler method for quantitative evaluation and automated scoring of immunohistochemistry (IHC) images [R2]. The CU-IScore algorithm assigns a score from 0-300 based on the staining intensity and distribution, with higher scores indicating stronger staining. By utilizing these quantitative scores, we ensure that our training stacks include images with a range of scores, creating a representative subset of the entire dataset for a given marker.

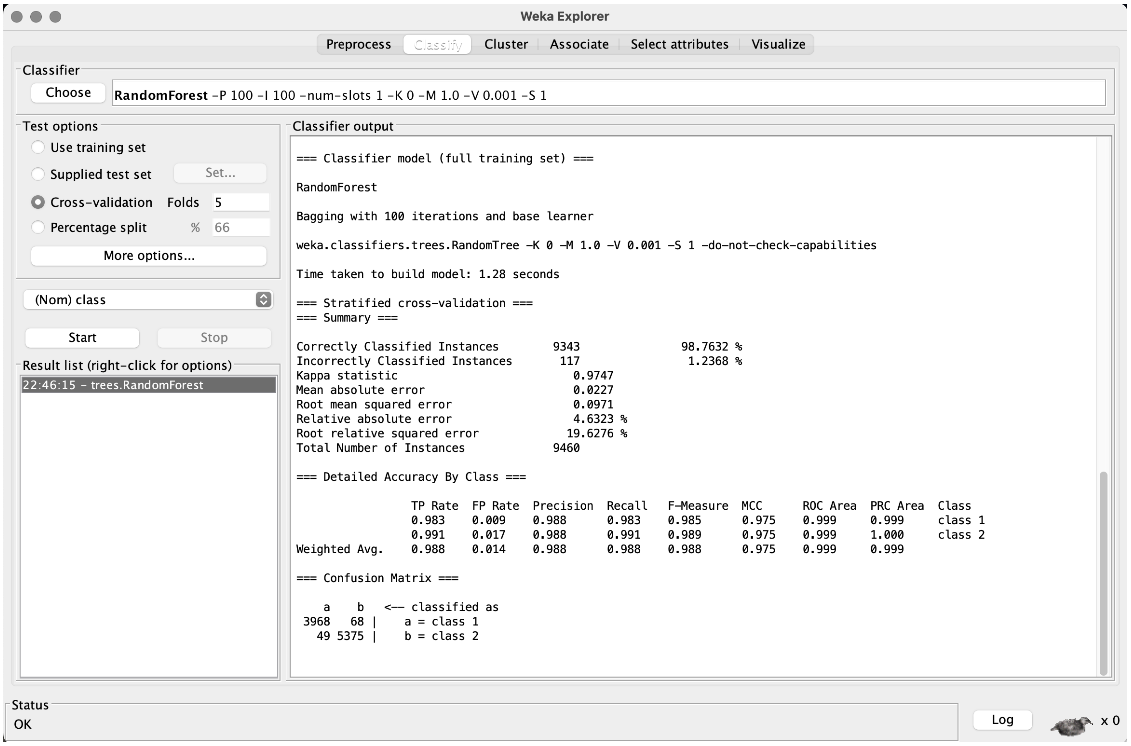

Fig. F1: **Built-in error analysis in WEKA for evaluating classifier performance.** Various error types, such as TP (true positive) and FP (false positive) rates, precision, recall, F-Measure (F1-score), Matthews Correlation Coefficient (MCC), ROC area (Receiver Operating Characteristic), and precision-recall curve (PRC) area are displayed. The results relates to CD20, a marker arbitrarily selected for demonstration.

Next, we use the Random Forest (RF) algorithm as a classifier and maintain the out-of-bag (OOB) error as a performance measure below a certain threshold. The OOB error is a performance measure calculated for the test data points that are not included in the bootstrap sample during the training process. Each decision tree within the RF algorithm is trained using a subset of the labeled data, with some samples held out from each tree's training process. The OOB error provides an estimate of the model's performance on unseen data. Additionally, we evaluation the model performance using multiple performance measures including areas under the receiver operating characteristic and the precision-recall curves to explore the sensitivity, specificity, precision, and recall of the trained classifier. Figure F1 shows the 5 folds cross-validation results of the models trained for CD20 marker. These performance measures can be easily generated during the training process to guide the user in building an appropriate classifier.

Once the classifier is trained, we apply the model to the remaining images from the same channel. As mentioned earlier, the training stack is selected randomly from images with high,

medium, and low CU-IScore values to ensure representation of the entire dataset. Finally, we visually inspect the generated FR maps to ensure that artifacts have been effectively removed from the images.

While the general training procedure remained consistent, for the two datasets used in this manuscript and given the number of images, we generated a stack using all the images in a channel rather than a subset of the data. However, we applied the trained model for certain markers to classify stacks from similar channels. For instance the model we trained for CD4 marker worked very well for CD8 channel.

We acknowledge the reviewer's suggestion that providing further details on our training and validation process would enhance the understanding of our framework. To address this comment we have extensively modified the "Method" section of our manuscript. Following lists the detail of modifications:

- The manuscript is modified on page 10 lines 291-328.
- Table S2 is added to the supplementary text to list all the extracted features and the information gain score that quantifies how informative each feature is in the classification task. This table is included in this response as Table T2.
- All models and data generated for all markers in both datasets are made available on the GitHub repository dedicated to this manuscript.

Major comments#2- (Part#1.) Fig 2: With respect to the general comment above, it is unclear for all panels what the training was done on. Where these images trained individually, were they part of a training set or were they part of a test set? (Part#2.) In panel A it does not seem that 163 shows cross-talk in 164, meaning the pattern of 163 cannot be seen in 164. It is also unclear what exactly the purple arrows point at (similarly for panels B and C). Why do the authors believe that Vimentin expression can be observed in the SMA channel? (Part#3.) In panel D, again, the authors should clarify the process of how the FR map was generated for this specific image. If the density of pixels is taken into account, then I would still expect to see the signal from cell C propagate to the FR map. However in the FR map there is no visible signal any more. The authors should clarify this.

We would like to thank the reviewer for these valuable comments. To ensure that we have addressed this comment appropriately, we have added part numbers to the text. We did not alter the order or content of the comments, but rather added part numbers as a means of ensuring

that all concerns have been adequately addressed.

Major comments#2- Part#1: Fig 2: With respect to the general comment above, it is unclear for all panels what the training was done on. Where these images trained individually, were they part of a training set or were they part of a test set?

All the example images used in Figure 2 of the manuscript were individually trained to demonstrate the process, but the measurements were performed on pixels that were not annotated or labeled. In other words, when measuring a specific cell (such as cell A or cell B), that particular cell was not included in the training data (labeled data). To provide clarification on the generation of the FR maps shown in panels a-f of Figure 2, we demonstrate intermediate steps involved in generating Figure 2 we have modified the caption of this figure to include the fact that the images are trained individually.

Major comments#2-Part#2: In panel A it does not seem that 163 shows cross-talk in 164, meaning the pattern of 163 cannot be seen in 164. It is also unclear what exactly the purple arrows point at (similarly for panels B and C). Why do the authors believe that Vimentin expression can be observed in the SMA channel?

Mass-spectrometry imaging techniques are are susceptible to channel crosstalk, which can arise from various sources such as impurities in the heavy metal ion source used for antibody labeling, modification of heavy metal ions, or gold ions from the slide surface [R4]. Crosstalk artifacts can occur, especially when comparing channels with similar atomic masses within $M \pm 1$ (due to impurity in mass isotope) or within $M + 16$ (due to oxidation). To identify channels prone to channel crosstalk, we refer to Table T1 [R3].

Based on the values in Table T1 we have enough evidence to visually inspect the SMA channel with a mass isotope of 164 for signal contamination from Vimentin channel with a mass isotope of 163. The inspection results confirm the presence of crosstalk for two main reasons: first, part of the signal does not follow the pattern/structure we expect for SMA; second, aside from the background noise, we observe two levels of intensities. One exhibits a high intensity signal, representing the SMA signal, while the other demonstrates low intensity values originating from the Vimentin channel.

To provide clearer demonstration, we present a new figure (see Fig. F2) with a clearer demonstration of channel crosstalk in which hepatocyte antigen staining (with M =145) in liver tissue is detected in the $M + 16$ channel (CD20 with M = 161). The generated FR map shows

TABLE T1: The crosstalk matrix is computed using single-stained beads [R3]. Diagonal values are set to 1, indicating no crosstalk within the same channel. By default, crosstalk is calculated only for potentially affected channels, including M±1, those associated with known isotopic impurities, and M+16 (Figure S2-d). The values within the cells represent the percentage of crosstalk from channels in the rows to channels in the columns. The numbers in the last column represent the total signal received in the respective channels.

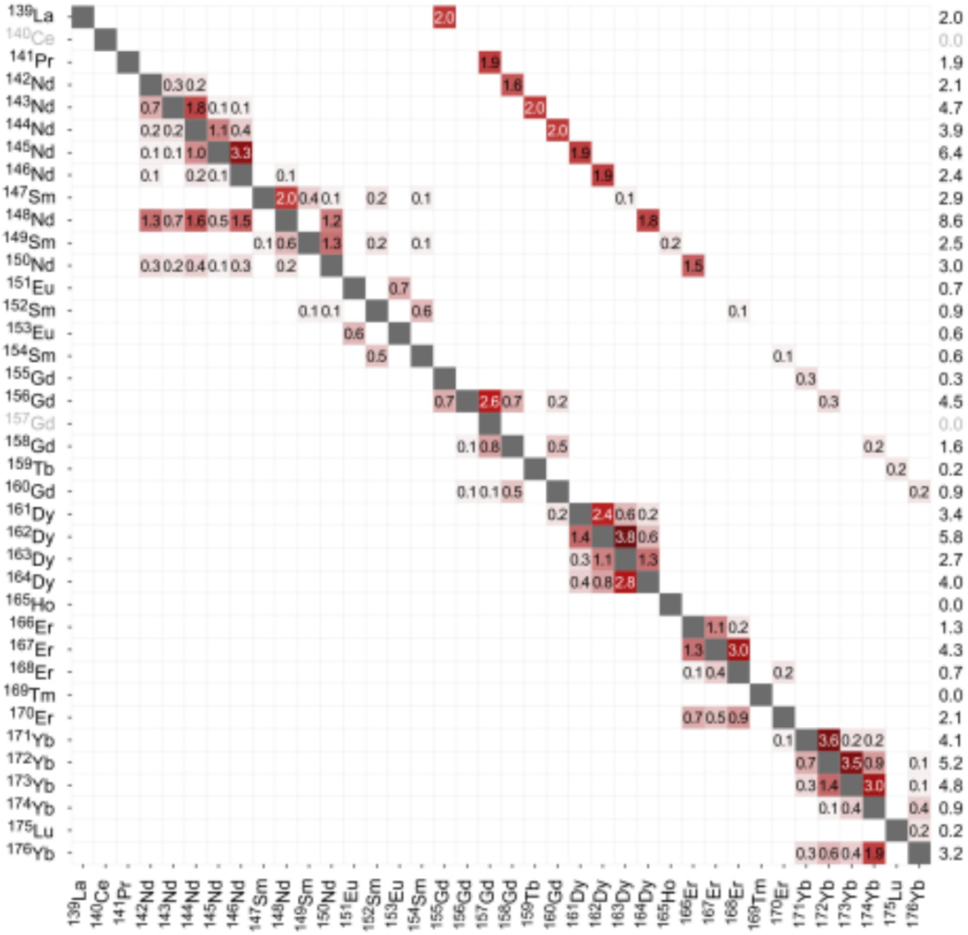

high probabilities for CD20 signal and medium probabilities for the crosstalk signal. In the subsequent step, by annotating the crosstalk pixels as artifact, we successfully eliminated all contamination from the final FR map. We note that in the second annotation we provided the classifier with only one more sample where pixels exhibited low intensity values and followed the pattern observed in hepatocyte antigen channel rather than the expected membraneous stain for CD20.

Major comments#2-Part#3: In panel D, again, the authors should clarify the process of how the

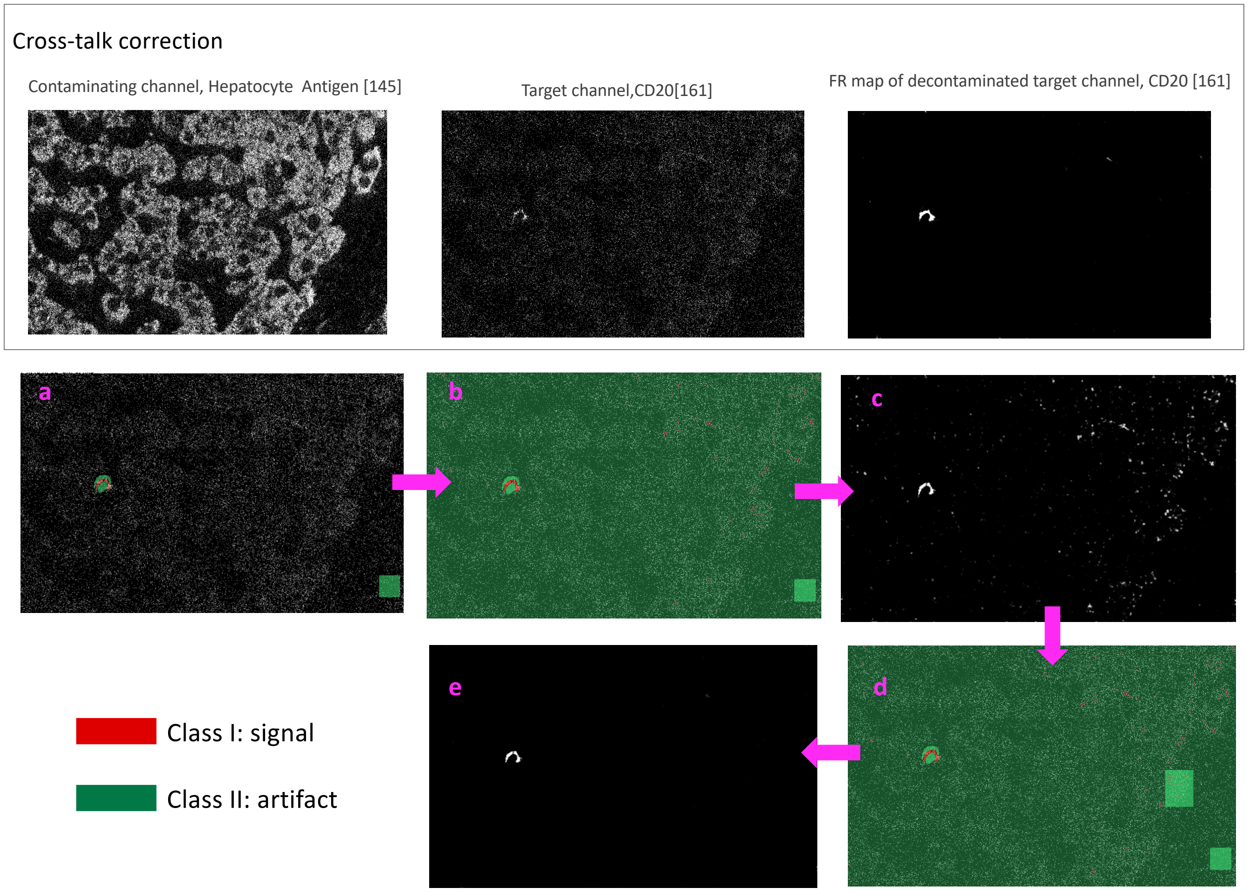

Fig. F2: **Intermediate steps involved in generating Figure 2-a in the manuscript.** An example of cross-channel contamination where a contaminating channel, Hepatocyte Antigen with mass isotope of 145 (top left), contaminates a target channel, CD20 with mass isotope of 161 (top middle). Our approach removes this contamination by generating an FR map for the target channel (top right). **a,** CD20 signal and salt and pepper noise are annotated as label data for class I and II. **b-c,** Result of classification and the corresponding FR map that includes medium expression values for certain pixels that exhibit crosstalk contamination. **d,** Crosstalk is labeled as artifact. **e,** The contamination resulting from crosstalk is effectively removed.

FR map was generated for this specific image. If the density of pixels is taken into account, then I would still expect to see the signal from cell C propagate to the FR map. However in the FR map there is no visible signal any more. The authors should clarify this.

To provide a more detailed explanation of the generation of Figure 2-d, we replicated this figure and included all annotations and intermediate FR maps. In the initial step, we classified the CD163 signal as class I (desired signal) and labeled only the salt and pepper noise as class II (artifact). However, the resulting FR map displayed high probabilities for the aggregate pixels, which were contamination rather than actual signal. In the subsequent step, we specifically

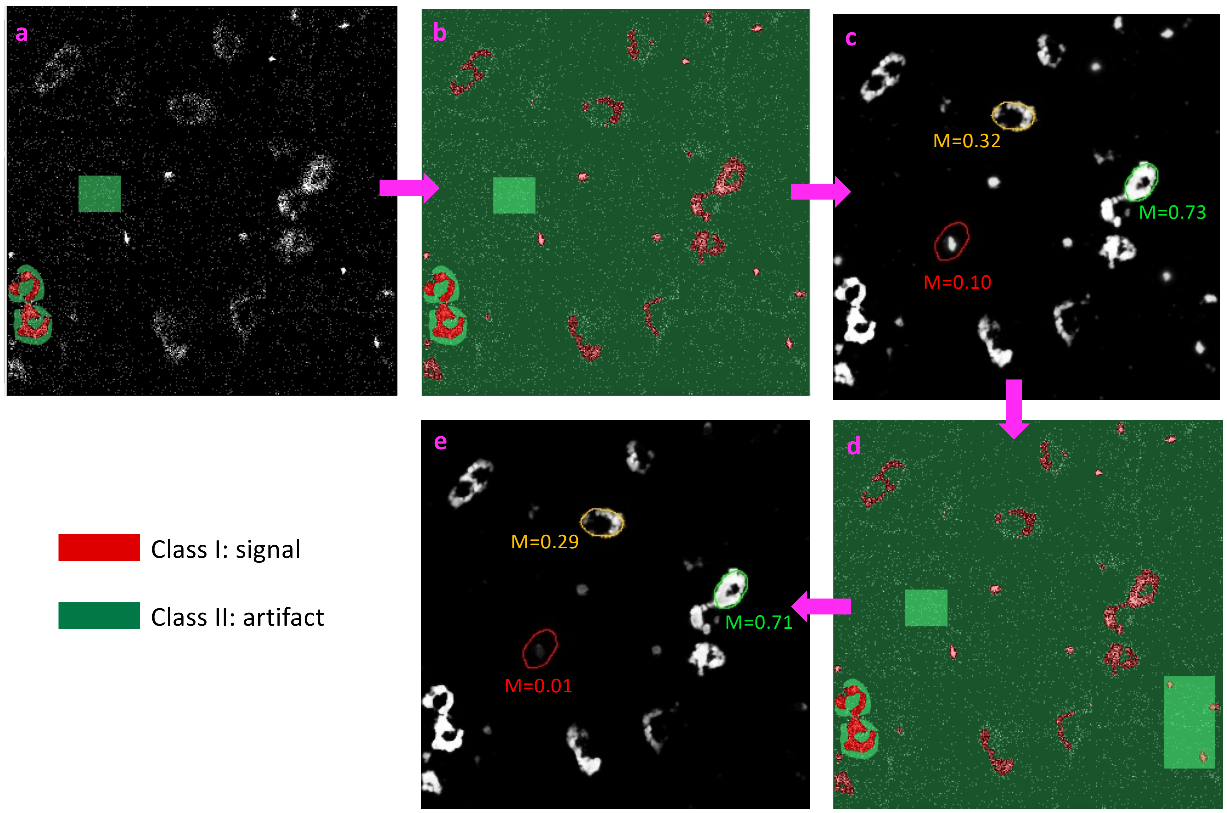

Fig. F3: **Intermediate steps involved in generating Figure 2-d in the manuscript. a,** CD163 signal and salt and pepper noise are annotated as label data for class I and II. **b-c,** Result of classification and the corresponding FR map that includes high expression values for certain pixels that exhibit aggregates. **d,** Aggregates which have very high intensity compared to real signal are labeled as artifact. **e,** The contamination resulting from aggregates is effectively removed.

labeled the aggregate pixels as artifacts since they exhibited significantly higher intensity values compared to the CD163 signal. This labeling adjustment effectively reduced the probability of those pixels belonging to the signal class and, consequently, removed or corrected the contamination from the final FR map.

To address all three parts of this comment, we have made the following modifications to the manuscript:

- The previous panel a of Figure 2 has been replaced with a more illustrative example of channel crosstalk. The caption of Figure 2 is updated accordingly.
- The caption of Figure 2 is modified to include the fact that all the images in this figure were trained individually.
- Manuscript is modified on page 4 lines 135-139 to describe the changes in Figure 2-a.

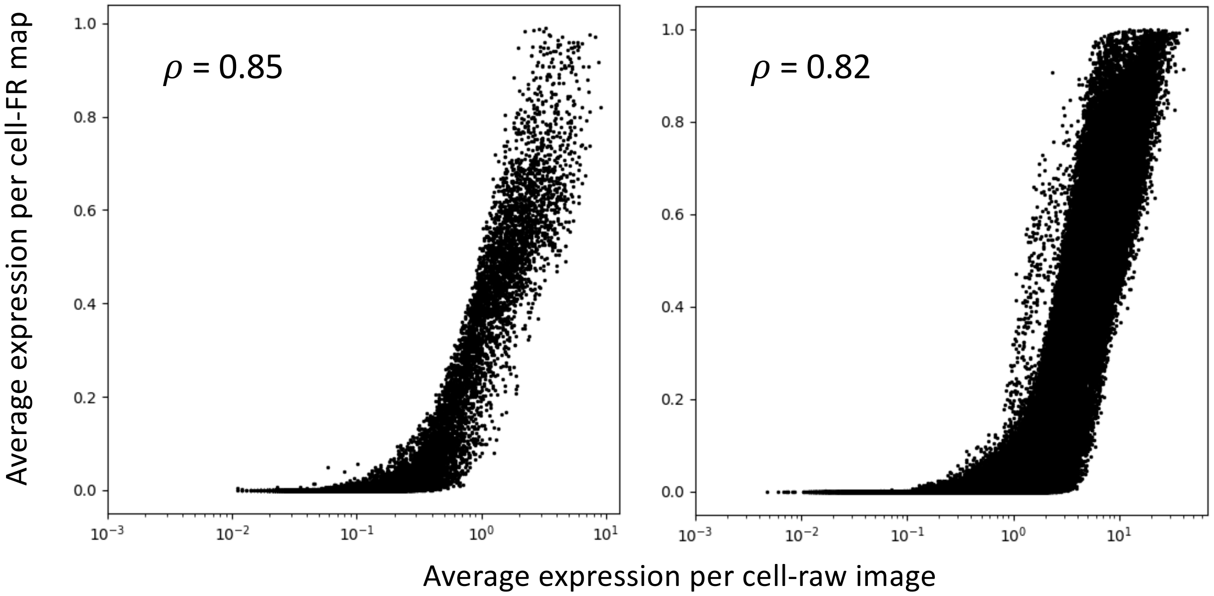

Fig. F4: **Correlation of Ki67 expression levels per cell as a functional marker.** The correlation between the expression levels of the Ki67 marker per cell is depicted using raw images (x-axis) and FR maps (y-axis) for ovarian cancer tissue obtained through fluorescence-based imaging (left), and for kidney tissue obtained through MIBI as an example of mass spectrometry imaging. Each subplot displays the Spearman's rank correlation coefficient.

Major comments#3- The authors show in Fig. 3a (and 4a) the correlation between the original raw values and the FR map values. Please provide rank based correlation coefficients. The question is, if the FR map, which is a mere probability of a pixel classified as signal or noise, is correlated with the original raw values. If not one needs to be carefully when comparing expression levels for functional markers such as Ki67. Please also provide the correlation for Ki67. If no high correlation is given (0.8) I would suggest that the authors should clearly state this limitation in the manuscript and point to the validity of phenotyping but limitations regarding expression level comparisons.

Following the reviewer's suggestion, we have now calculated the Spearman's rank correlation coefficient for all definitive markers within both the ovarian cancer and breast cancer datasets. We have included these coefficients in the correlation plots within the manuscript and also in the supplementary text.

Furthermore, as recommended by the reviewer, we specifically computed the Spearman's rank correlation coefficient for the Ki67 marker as an example of functional markers. The corresponding correlation plots can be found in Fig. F4.

While this correlation coefficient demonstrates that our method can still be employed for evaluating functional markers, we acknowledge the challenge posed by the low counts and pixelated nature of the signal in mass spectrometry imaging technologies. Given the current state of the technology, it is difficult for us to envision the assessment of functional marker expression levels, regardless of the methods or analysis pipelines employed to analyze imaging data.

To address this comment we have revised our manuscript as follow:

- Figure 3-a on page 7 is modified where the Spearman's rank correlation coefficient is added to each correlation plot. The caption of this figure is updated accordingly.
- Manuscript is modified on page 6 lines 201-202 to describe the changes in Figure 3.
- Figure 4-a on page 8 is modified where the Spearman's rank correlation coefficient is added to each correlation plot. The caption of this figure is updated accordingly.
- Figure S1-a is modified where the Spearman's rank correlation coefficient is added to each correlation plot. The caption of this figure is updated accordingly.

Major comments#4- Please provide statistics throughout the manuscript when arguing for significance.

To address this comment, we have calculated multiple statistical measures to quantify the qualitative illustrations.

- Pearson correlation coefficients are calculated on patient level to quantify the correlation between our identified cell types versus the baseline (see Fig. F5).
- Scatter plots in Figure 4-b and Figure S1-c is replaced with a confusion matrix that quantifies the cell-cell comparison between our identified cell types versus the baseline in the ovarian cancer dataset.
- Spearman's rank correlation coefficient is calculated for all definitive markers in both datasets to quantify the correlation between marker expression per cell measured from raw images versus the FR maps. The coefficient is displayed on the corresponding subplots in Figures 3-a, 4-a, and S1-a in the manuscript.

Major comments#5- There seem to be duplicated references. E.g. 21 and 27, or 19 and 26. Please correct.

We addressed this issue by removing the repeated references 26 and 27.

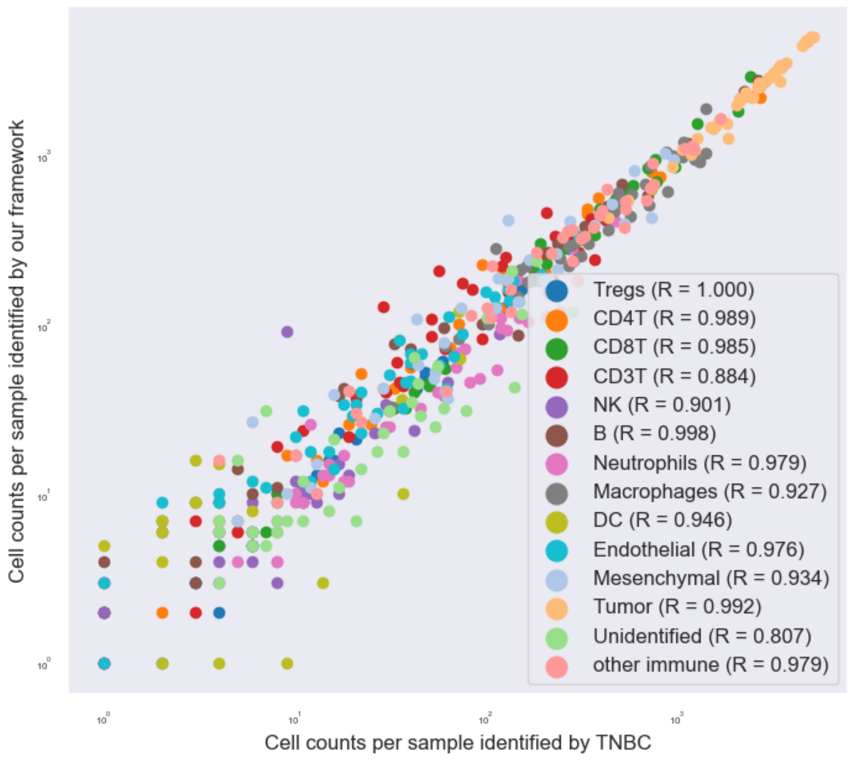

Fig. F5: **Strong correlation in the counts of different cell types identified on a patient-level in the TNBC study and using our framework.** Correlation between the frequency of each cell type per patient identified using MAUI in the TNBC study (x-axis) and our proposed framework (y-axis). Pearson coefficient is calculated for each cell type for the 41 patients in the study.

Minor comments#1- The authors should provide single cell heatmaps depicting the FR map expression of the detected cell types for both datasets to see the result of the clustering. Displaying a sub-sample of the data should be fine (e.g. 10000 cells).

Following the reviewer's suggestion, we have generated heatmaps for both the ovarian cancer and breast cancer datasets, as depicted in Fig.F6. The heatmaps display the average expression per cell for the definitive markers (x-axis) across the cells (y-axis) in the datasets.

To facilitate a comparison, we included a heatmap generated using InForm cell type identification results (Fig.F6-a-left) next to the heatmap that demonstrates the results for our framework (Fig.F6-a-right) for the ovarian cancer dataset. This allows for a visual assessment of the disparities and improvement offered by our framework compared to the baseline.

Similarly, we produced a corresponding heatmap for the breast cancer dataset (see Fig.F6-b), which shows the expression levels of definitive markers per cell for the identified cell types. To address this comment, we made the following modifications to the manuscript:

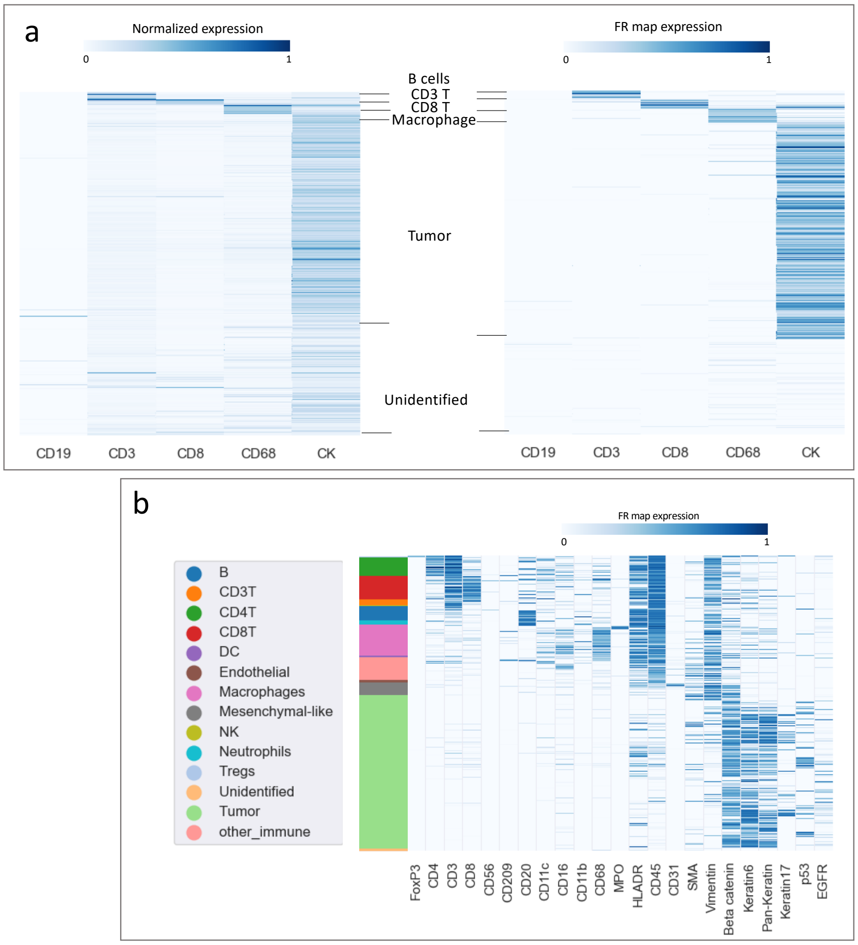

Fig. F6: **Cell types clustered by marker expression**. Expression values for each marker are measured from the raw image (left) and the FR maps (right). Stacked bar plot shows the abundance of each cell type in the breast cancer dataset.

- We appended a figure to the supplementary text (Figure S2) to present the heatmaps for the ovarian cancer dataset.
- We annotated the heatmap for the breast cancer dataset with cell types and incorporated it into Figure S1 in the supplementary text (see Figure S1).

Minor comments#2- Fig. 3c: unclear why "predicted" cell types? Are these not simply the cell counts based on phenotyping?

We agree that the axis label should be modified and we replaced "predicted" with "identified". To address this, the following changes have been applied to the manuscript:

- The labels of x and y-axis of Figure 3-c are updated.
- The labels of x and y-axis of and Figure 4-b are updated.

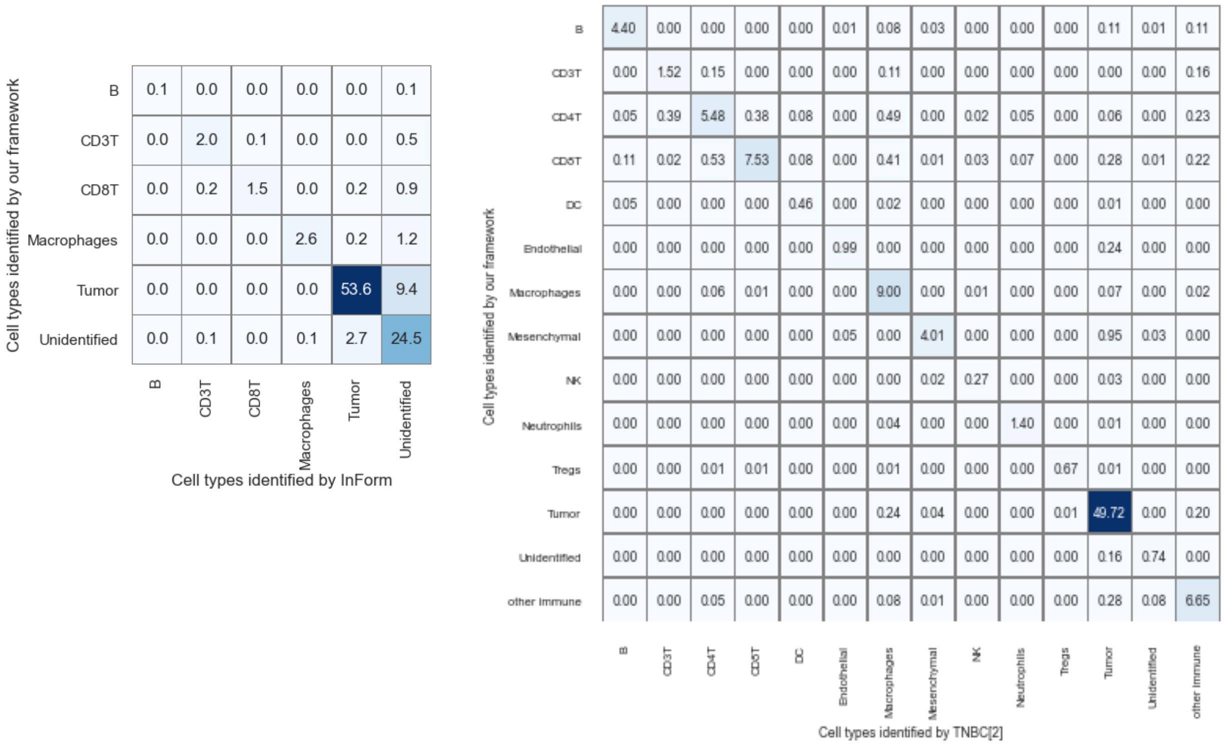

Fig. F7: **Comparison between the cell types identified in the TNBC study versus our framework.** Table entries indicate the percentage of cells in the dataset where columns and rows, respectively, compare their identified types by the baselines and our framework.

- The labels of x and y-axis of Figure S1-c are updated.

Minor comments#3- In Fig. S1C and Fig. 4b the authors should provide a better display of the data for comparison. The values of the confusion matrix should be shown instead of the black dots.

We replaced each scatter plot in Figures 3 and 4 by a matrix where the entries list the percentage of cells in the dataset and rows and columns are, respectively, the cell types that we identified versus the baselines (see Fig F7).

To address this, we have applied the following modifications to the revised manuscript:

- The scatter plot in Figure 4-b is replaced with a matrix and the caption of the figure is modified accordingly.
- Manuscript is modified on pages 7-8 lines 221-226 to account for the change above.
- The scatter plot in Figure S1-c is replaced with a matrix and the caption of the figure is modified accordingly.
- Manuscript is modified on page 7 lines 211-212 to account for the change above.

*Minor comments#4- In the discussion the authors state that the approach requires less hands on time but is more computationally intense. However, it is unclear to me if manually labelling 40 channels can be considered low manual input. If I calculate correctly, then labelling 40 channels takes 40*10 min   5 hours. On top there will be roughly 4000 mins (3 days) of FR map calculation for a fairly small dataset of 41 images. With respect to expert knowledge, do the authors believe that labelling signal and noise for 40 channels requires less knowledge than thresholding or other manual curation steps? So please specify if by "high runtime" you only mean computational or also hands on time and clarify in the text and potentially indicate that labelling images also requires some expert knowledge.*

We appreciate the reviewer's comment and have taken it into consideration. In our pipeline, we divide the computational time of data analysis into three major parts: 1) generating labeled data, 2) computing FR maps, and 3) clustering cells and identifying cell types. We define the hands-on computing time as the sum of steps 1 and 3, and our pipeline reduces this hands-on time.

The time required for labeling training data ranges from 10-15 minutes, depending on the variability of signal in different channels of a dataset. For example, 10 annotations per class are sufficient for a marker such as Foxp3 (which is not frequently expressed), while more examples are needed for more ubiquitous signals like Beta-tubulin or Vimentin. Notably, the numbers we reported are worst-case scenarios where we need to build one model per channel/marker. However, in many cases when the signal properties of markers are similar, models built for one marker can be transferable to the other similar channels. For instance, the model built for CD8 can be used for CD3 and CD4 channels. Similarly, the model built for CD31 is transferable to Podoplanin, and the Keratin6 and Keratin17 channels can also be trained with the same models. This approach reduces the need to build models from scratch, and we may only need a few correcting annotations, which requires less time than building a model from scratch. Thus, the hands-on time needed in practice is often less than what we reported.

Moreover, our pipeline's enhanced signal-to-noise ratio and normalized data quality result in faster downstream clustering. We perform the clustering in multiple iterations, overlay the identified cell types back to the images, and manually inspect their accuracy for quality control. We repeat this process until we are confident that there is no systematic error in identifying cell types. Our observation shows that this quality control process is faster when using FR maps

instead of raw data. While it is challenging to measure the time we save in quality control step and using using models that generalize across similar channels, we assert that the hands-on time required for our pipeline is less than that of existing pipelines such as MAUI which removes step by step removing one artifact at each step.

To address this, we have applied the following modifications to the revised manuscript:

- We have modified our manuscript on page 9 line 9 lines 266-267 to indicate the expert knowledge that is required for labeling. Lines 274-284 explains the computational complexity of our approach.

Minor comments#5- The authors should add this paper to introduction and potentially discuss differences and advantageous of their approach. PMID 34196108

We would like to thank the reviewer for suggesting this reference. In the suggested paper, the authors have employed two normalization techniques to account for variations in immunodetection signal intensity between samples. The first method involves manual background identification, which requires the user to visually inspect each marker and set a minimum intensity/mean threshold to remove background noise. Using the MCD viewer, the user-defined threshold is identified, and the pixel values are then binarized so that all pixels below the threshold are set to 0 and all pixels above the threshold are set to 1. Normalized cell intensities are then defined as the frequency of positive pixels per cell.

The second method, semi-automated background identification, employs supervised pixel classification using ilastik software, where the Random Forest algorithm assigns each pixel to either ”signal” or ”background” based on user-defined training inputs. The images are then binarized using a user-defined threshold, and the relative frequency of positive pixels in a cell is measured on the cell mask.

While especially the pixel classification is very similar in addressing the removal of artifacts, the output differs significantly. Our approach retains more information for the downstream analysis by avoiding binarization of the input data. To address this comment we have applied the following modifications to the revised manuscript:

- We have cited PMID 34196108 (reference # 35) and modified our introduction on page 2, lines 63-67 to refer to this reference.

Response to Reviewer 2

The authors make a strong case that pixel classification is a good approach to combine several steps of noise and artifact removal into one step. They demonstrate how applying this to multiplex tissue images can provide pixel values that are good for downstream analysis. This is an important result and will give confidence to other researchers to follow this approach, thereby enabling the possibility of having a significant impact on the field of multiplexed image processing. While this idea may occur to other people, the paper provides a useful contribution in showing that this is an effective technique by providing detail of the transformation as well as showing that it performs well in real biological analysis problems. The authors make a clear case as they state "The pixel density should be considered along with the intensity." and show this point throughout the paper. The authors also talk about how their technique performs normalization, and I have some concerns about this, and give more details in major issue 1. My overall impression is that it is a quality, clear and useful paper, though I do have a major concern that should be addressed before publication.

We thank the reviewer for all of the constructive comments and suggestions.

Major issue#1- One major issue is how the authors talk about the normalization their algorithm performs, and I am concerned about its relationship to quantitative downstream analysis. One of the main motivating factors in this work is to provide normalization. This is evidenced in the abstract where 'normalized pixel values' are mentioned as well as on page 2: "Therefore, high-quality imaging data should ideally satisfy the following criteria: 1) the data should exhibit high signal-to-noise ratios (SNR) and be as free as possible from artifacts; and 2) be appropriately normalized to remove non-biological signal variability within and across acquisition batches and tissues." and also on page 2: "Our approach combines denoising and removal of various artifacts into a single pixel classification step, outputs a feature representation map (FR map) that eliminates the need for any further normalization process". Normalization often refers to a process where the mean or maximum value of some pixels are shifted, but that preserves the relationship between pixels such that pixels that are brighter in the original image are brighter in the processed image. Normalization can also be an appropriate way to refer to batch correction as the authors do on page 2: "be appropriately normalized to remove non-biological signal

variability within and across acquisition batches and tissues". This statement seem correctly supported by the work, as the normalization they perform does seem to occur over batches and tissues, as is desirable. However, from what I can see, the 'normalization' in this work also 'normalizes' between different cells within one image, so pixels that are part of class 1 'signal' are not normalized in a way that is desirable for most downstream analysis. Within one image, if pixel A and pixel B are part of the signal class and pixel A is brighter than pixel B in the original image, then it is possible than pixel B becomes brighter than pixel A in the processed image. To put this in more mathematical terms, the mapping within the signal class of pixels in not monotonically increasing and this distortion of pixel values in the final image could provide problems in any cell-level downstream analysis. One example of this distortion of class I pixels can be seen in Figure 2d. Consider the cell in the bottom middle of the image, which I will call cell D (location is horizontally between B and C, and vertically much below both of them). If you compare the brightness cell D compared to A before and after: before cell D is much darker than cell A, and afterwards cell D and cell A look about equal brightness. This loss of distinction could negatively impact quantitative analysis of the signal. Another way to see this is in the graphs in figure 3a. For example, in the CD3 case, some pixels that raw image value of about 0.5 map to the FR map with a value of 1, but some pixels with a raw image value of 2 have an FR map value of 0.8, inverting the brightness of the original values. This might be pixels from different images, but it is not possible to tell from the data presented and so there remains a concern about how the normalization modifies values within the signal class within one image. I can think of two ways to address major issue 1. 1. A graph of pixel intensities of the pixel in class 1 (or above 0.5 in class 1). This graph would be similar to the graphs in Figure 3a, but should be split by different origin image so that it is possible to see whether higher signal pixels in the original image produce higher signal pixels in the processed image. It may be helpful if this graph was not log scale. If a graph like this show that signal is mostly monotonically increasing within each image, then that would remove this concern. 2. Description of some of this issue in the discussion, highlighting the non-linearity within the signal that is created, acting as a warning to those who would do downstream analysis on the FR map.

We would like to thank the reviewer for this insightful comment. We agree that further discussion regarding this aspect is necessary. To address this comment, we will start by quantifying the nonlinear conversion from raw images to FR maps. Additionally, we will provide clarification

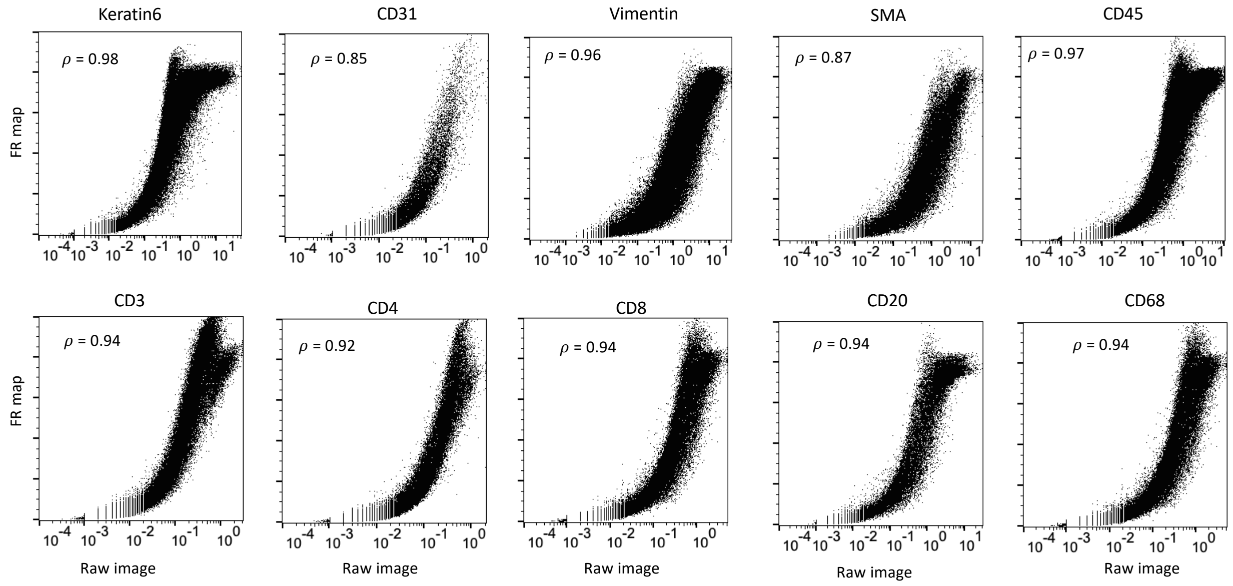

Fig. F8: **Correlation of expression level per cell for selected markers displayed in Fig.3a.** Correlation between expression level of markers per cell from raw images (x-axis) and FR maps (y-axis) is shown for multiple markers from breast cancer dataset. Spearsman's rank correlation coefficient is displayed on top left.

on the steps involved in generating the FR maps, with specific emphasis on Figure 2-d as an illustrative example.

To quantify the nonlinear relationship between pixel values in raw images and FR maps, we have calculated the Spearman's rank correlation coefficient for all definitive markers in both the ovarian cancer and breast cancer datasets. The results of this analysis have been presented in the correlation plots within the manuscript and the supplementary text. Figure F8 displays a subset of correlation plots, including the calculated rank correlation coefficient, which confirms the nonlinear yet monotonically increasing relationship between the intensity values of the raw images and the FR maps.

Next, we clarify why the hypothetical cell called "cell D" that exhibits bright values in the original image but reduced illumination or intensity values in the FR map. To visualize this we present the intermediate steps involved in generating the FR map in Fig. F9. First, we annotate the salt and pepper noise as artifact and several examples of positive signal. In the FR map at this step, aggregates pixels have high probability. It is important to note that the pixels in "cell D" are considered artifacts and can be mistakenly identified as signal or positive cells if not removed. Aggregates refer to small collections of very bright pixels that do not align with the expected staining structure for a marker. Next, we add labels that annotates aggregates as artifact

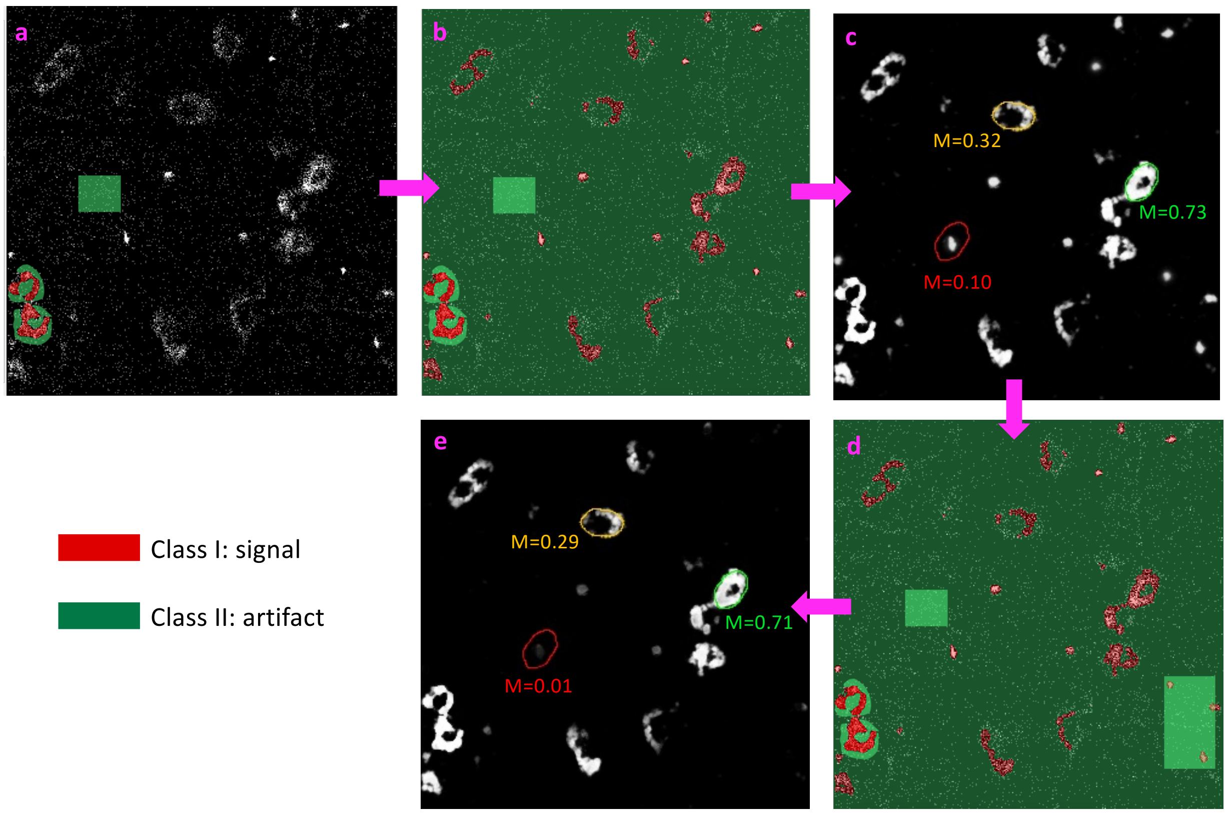

Fig. F9: **Intermediate steps involved in generating Figure 2-d in the manuscript. a,** CD163 signal and salt and pepper noise are annotated as label data for class I and II. **b-c,** Result of classification and the corresponding FR map that includes high expression values for certain pixels that exhibit aggregates. **d,** Aggregates which have very high intensity compared to real signal are labeled as artifact. **e,** The contamination resulting from aggregates is effectively removed.

(see F9-d), as a result their probability in the final FR map is reduced significantly.

It is important to note that this artifact removal process affects pixel values in a nonlinear manner. Therefore, a bright pixel in the raw image may not appear as a bright pixel in the FR map if it exhibits artifact, such as the showcase for the aggregates. Similarly, a pixel with a medium value may not appear with a medium intensity in the FR map if it stems from a contaminating channel causing crosstalk. However, except for the artifact pixels, the conversion from the raw image to the FR map is nonlinear but monotonically increasing as shown in F8. To address this comment we have revised our manuscript as follow:

- Figure 3-a on page 7 is modified where the Spearman's rank correlation coefficient is added to each correlation plot. The caption of this figure is updated accordingly.
- Manuscript is modified on page 6 lines 201-202 to describe the changes in Figure 3.

- Figure 4-a on page 8 is modified where the Spearman's rank correlation coefficient is added to each correlation plot. The caption of this figure is updated accordingly.
- Figure S1-a is modified where the Spearman's rank correlation coefficient is added to each correlation plot. The caption of this figure is updated accordingly.

Minor issue #1- A related issue within this is that the paper does not seem to describe what exactly the feature representation quantifies. It may be the probability of being within a class, or that probability multiplied by original signal, or something more complex. An explanation or a link to where this is explained is essential for helping to deal with this issue.

The output of the classifier consists of two FR maps, one for each class. The pixel values in each FR map represent the probability of that pixel belonging to the corresponding class. Pixels with noise and artifacts have either zero or very low values in the FR map of class I, meaning FR maps of class I are free from noise and artifacts. To address this comment we have added a reference to the TWS manual in the manuscript on page 4 lines 117-118.

Minor issue #2- In the abstract 'pixel-accurate representations' could be changed to 'pixel-resolved representations' or 'pixel-resolution representations' or 'pixel-scale representations' or 'pixel-precise representations' or something similar to make it clearer that you are not talking about the accuracy of pixel values, but are making a scale/size claim.

We would like to thank the reviewer for this suggestion. We agree that using the term "pixel-accurate" can have unintended implications for accuracy. Therefore, we have modified our manuscript to replace "pixel-accurate" with "pixel-scale," which better describes our intent.

- The manuscript is modified on page 1 line 26.

Minor issue #3- In the introduction you mention (page 2) "user-defined thresholding parameters that are prone to bias". This is fine, but it may be worth adding a discussion point about how your method needs training data, and that training data can be prone to some bias too.

We agree with the reviewer that annotating images with examples for signal and noise requires expert knowledge and this can be the source of introducing bias to the data.

We have modified our manuscript on page 9 lines 266-271 to further explain about the required expert knowledge.

Minor issue #4- On figure 3, the FR map y scale values should be quote as '0.8' not '0.800'.

We would like to thank the reviewer to bring this typo to our attention. To address this comment we have fixed this issue and replace the first plot of Figure S1-a with the same plot where the axis values are corrected. We have also noticed similar inconsistency in values of y axis in Figure 4-a and fixed that as well.

- Manuscript is modified on page 8, Figure 4-a where the values of y axis in correlation plots are consistently listed.
- The supplementary text is modified, Figure S1-a where the values of y axis in correlation plots are consistently listed.

Minor issue #5- On the issue of uniqueness, it would be worth describing how your work is unique against Giannakeas et al. "Segmentation of microarray images using pixel classification—Comparison with clustering-based methods". This could include just having two classes, using multiplexed images, or other things.

We would like to thank the reviewer for bringing this reference to our attention. The paper mentioned proposes the use of pixel classification for the segmentation of microarray images and demonstrates that supervised pixel classification outperforms clustering-based methods. While the idea of classifying the pixels into two classes of signal (spot) and noise/artifact (background) is similar to our proposed approach in this manuscript, the difference lies in the fact that, at the final step, they generate a mask for the image where the signal has the value of 1 and noise has the value of -1. However, in our proposed approach, we use the generated FR map without thresholding and demonstrate that using the FR map instead works well for the downstream analysis of cell-type identification.

We have introduced such techniques in our manuscript's introduction section and we modified our manuscript to include the reference introduced by the reviewer.

- The manuscript is modified on page 2 lines 63-67.

Other points #1- The introduction is extremely clear and well-motivated, particularly the first paragraph. The document is also filled with very helpful summary statements including: "For instance, while fluorescence-based imaging data can be denoised using intensity-based methods, MIBI data require density-based methods for denoising.". This statement helps explain why the proposed method is suitable across multiplexed imaging types.

We would like to thank the reviewer for these comments and aim to address their specific

points to ensure further clarity.

Other points #2- The authors state that data is available on request. This is acceptable in my opinion, though the authors may want to consider making the data publicly available.

We would like to thank the reviewer for this comment. we would like to confirm that the ovarian cancer data used in this study, which was collected in-house, is available in the associated Github repository for this manuscript. We have taken the necessary steps to ensure that both the trained models and the generated data from the model training process are included in the repository. Regarding the other datasets utilized in this manuscript, we have provided references to these datasets within the manuscript itself and the associated Github repository. This way, we have made sure that readers and researchers have the required information to access and retrieve those specific datasets.

Response to Reviewer 3

We would like to thank the reviewer for providing us with insightful comments. To ensure that we have addressed each comment appropriately, we have added comment numbers to the text. We did not alter the order or content of the comments, but rather added comment numbers as a means of ensuring that all concerns have been adequately addressed.

(comment #1.) This manuscripts makes an attempt at establishing a sought after "framework" or "pipeline" for the analysis of multiplex fluorescence and use it for cell type discovery. However it falls onto a rebranding problem. This work is not platform independent it uses a series of modules that already exist and are in use and depend on the platforms they are installed on. (comment #2.) The authors often mention the many existing technologies around mIF but compare to very few and a closed one, inform. I suggest reading the review by Parra et al [1] it might also be good to reduce unnecessary references. (comment #3.) The authors claim that they have found a way to deal with intensity normalization issues by segmenting (which they rebrand as "FR maps"). However the segmentation is done with the well known Ilastik whose features do not include intensity insensitive features, making intensity normalization still a problem. With the segmentation there is still a threshold (or several, in a tree) to be made amongst features that are sensitive to intensity changes. (comment #4.) In Solorzano et al [2] an attempt is done to overcome this problem with the introduction of an intensity insensitive feature and the introduction of deep learning features, even a segmentation is also done. (comment #5.) In [3] the QuPath software is used and it combines what is done here (in a single and truly platform independent framework with a friendly user interface) by extracting features like Ilastik (plus deep learning features) and using Random Forests and also Deep learning to do segmentation. I am aware that there is a difference between the amount of channels available in the data used in [3] and this manuscript but the platform works for both. (comment #6.) [2] also compares with CODEX data. Why not compare your methodology with your references 2 and 4 (Leet and Goltsev)? Comparing with InForm is not very informative unless you happen to own an Akoya device, but I do like the comparison with MAUI. I would still like to see a comparison with QuPath. In general, classical image features have been shown to not be enough to capture variety and deep learning features should be explored, how to do it is another story, it has to be

chosen by the researcher, all platforms, theoretical and software already exist, so in this aspect this manuscript doesn't bring anything new. Additionally Weka is regarded in the bioinformatics scene as a more exploratory tool but not useful for scaling to bigger experiments. Speaking of which, there is no comparison in terms of time and performance, any time I use InForm it is extremely slow, although it lets you do some parameter modifications, and parameters are also not shared or discussed. How are MAUI and InForm different/similar? (comment #7.) *When Random Forests are used, it is common to show feature importance, this way one can actually observe which features were more meaningful for the classification so this would be interesting to see. The overall idea it's still good, as a showcase of the use of simple, common and well known and used methodologies and stresses the importance of denoising and noise modeling and discusses the issue of normalization. So in summary the strength of this manuscript lies not in a novel or platform independent framework/pipeline but as a showcase of a commonly used set of methodologies and showing that it works. Data like the one used has already several studies so I am sure there are more instances to compare to.* (comment #8.) *Figure S1 does worry me a bit as there is what seems to be a big confusion with Macrophages and Tumor, which is actually common in MIBI and mIF data. While the plot looks nice, numbers would be more appreciated and easy to compare to.* (comment #9.) *A final comment is that at the time I finished this review, the repository's readme doesn't really explain what is contained in the rest of the code and was last updated 5 months ago with no modifications (even if it says "repo under development" there has been no development. At least the code has comments which is good. Having to separate the images into channels out side the code makes it an additional step that contributes to this work not truly being platform independent, there are many steps and requirements that are not really discussed.*

[1] Parra ER, Francisco-Cruz A, Wistuba II. State-of-the-Art of Profiling Immune Contexture in the Era of Multiplexed Staining and Digital Analysis to Study Paraffin Tumor Tissues. Cancers (Basel). 2019 Feb 20;11(2):247. doi: 10.3390/cancers11020247. PMID: 30791580; PMCID: PMC6406364.

[2] Solorzano L, Wik L, Olsson Bontell T, Wang Y, Klemm AH, Öfverstedt J, Jakola AS, Östman A, Wählby C. Machine learning for cell classification and neighborhood analysis in glioma tissue. Cytometry A. 2021 Dec;99(12):1176-1186. doi: 10.1002/cyto.a.24467. Epub 2021 Jun 22. PMID: 34089228.

[3] Viratham Pulsawatdi A, Craig SG, Bingham V, McCombe K, Humphries MP, Senevirathne S, Richman SD, Quirke P, Campo L, Domingo E, Maughan TS, James JA, Salto-Tellez M. A robust multiplex immunofluorescence and digital pathology workflow for the characterisation of the tumour immune microenvironment. Mol Oncol. 2020 Oct;14(10):2384-2402. doi: 10.1002/1878-0261.12764. Epub 2020 Sep 1. PMID: 32671911; PMCID: PMC7530793.

Comment #1. This manuscripts makes an attempt at establishing a sought after "framework" or "pipeline" for the analysis of multiplex fluorescence and use it for cell type discovery. However it falls onto a rebranding problem. This work is not platform independent it uses a series of modules that already exist and are in use and depend on the platforms they are installed on.

We thank the reviewer for this comment. We would like to clarify that when we described our analysis framework as platform-independent, we were referring to its independence from the imaging technologies used to collect the data, rather than the platform used to run the scripts or train the classifier.

Specifically, we have demonstrated that our framework can be used to analyze data acquired using both mass cytometry instruments, such as MIBI, and fluorescence-based imaging platforms, such as Polaris. Our intention is to address the challenges that arise from the specific characteristics of each imaging platform, such as signal-to-noise ratio and acquisition artifacts, during the image preprocessing step. As highlighted by [R5], there is currently no consensus on the most appropriate method to denoise images, and researchers tend to employ their own approaches based on the level and composition of the observed noise. This leads to customized solutions [R4], [R6]–[R8] developed for specific imaging platforms reducing the framework's applicability across different imaging platforms and hindering reproducibility and data integration. Our aim is to tackle this issue by approaching the preprocessing, denoising, and artifact removal as a pixel classification problem within a unified framework, utilizing previously developed software packages that can solve this problem consistently across different imaging platforms and technologies. In this context, our framework is platform-independent.

To address this comment we have modified our manuscript to make it clear that when we use the term "platform," we are referring exclusively to the imaging platform.

- The manuscript is modified on page 2 line 47.

- The manuscript is modified on page 6 line 186.

Comment #2. The authors often mention the many existing technologies around mIF but compare to very few and a closed one, inform. I suggest reading the review by Parra et al [1] it might also be good to reduce unnecessary references.

We would like to thank the reviewer for suggesting the review paper, which provides a comprehensive overview of recent advancements in multiplexed staining and digital analysis techniques for detecting immune cell types and their spatial distribution within tissues. The review paper also introduces widely-used analysis tools and software packages in this field. Although our manuscript focuses on specific datasets and platforms, we have utilized one dataset from a mass cytometry instrument (MIBI) and another from a fluorescence-based imaging instrument (Polaris) to demonstrate the applicability of our framework. Additionally, we have utilized software and tools mentioned in the review paper, such as ImageJ and InForm, along with MAUI, which was not covered in the review. To address this comment we have appropriately cited the review paper in our manuscript.

- The manuscript is modified on page 1 line 35.

Comment #3. The authors claim that they have found a way to deal with intensity normalization issues by segmenting (which they rebrand as "FR maps"). However the segmentation is done with the well known Ilastik whose features do not include intensity insensitive features, making intensity normalization still a problem. With the segmentation there is still a threshold (or several, in a tree) to be made amongst features that are sensitive to intensity changes.

We would like to thank the reviewer for this comment. We also would like to clarify that we did not use the Ilastik software to perform pixel classification. The Ilastik icon in Figure 1 is included only to mention that other software packages can be used for pixel classification and it is not limited to WEKA. We will remove these icons to avoid causing any future confusion for the readers. In addition, as we stated in the manuscript, the intensity alone does not have sufficient information for denoising mass spectrometry images. Spatial information of pixels, in addition to intensity, in effect the density of the pixel intensities are also included to assist in classifying a pixel as signal or noise. In fact, we used WEKA segmentation tool that includes features that are sensitive to spatial distribution of pixel values. Following are a subset of the features we extracted to train our classifiers:

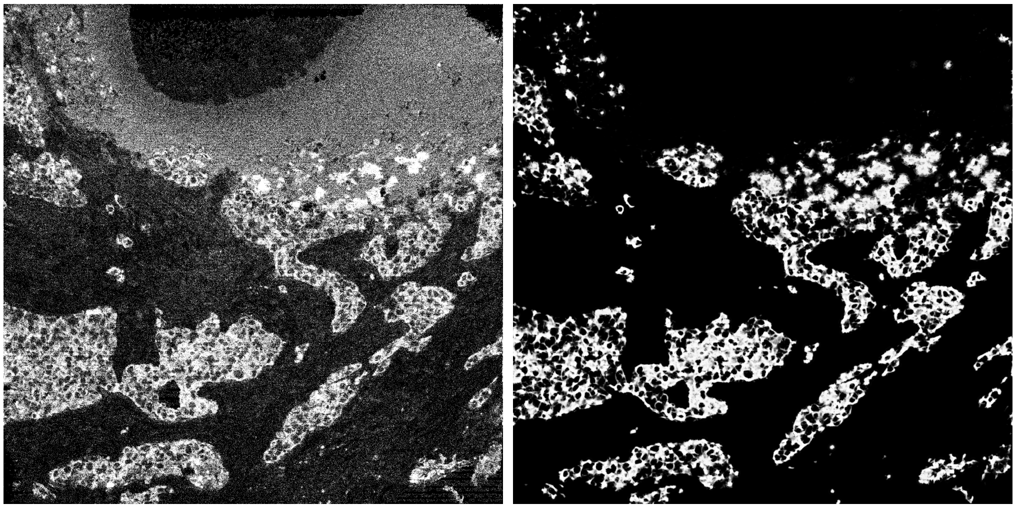

Fig. F10: **A comparison between raw image and FR map.** Section of breast tissue stained with Pan-cytokeratin antibodies before (left) and after (right) removal of backgrounf noise and necrotic tissue regions.

- Mean is calculated from the pixels within a radius of σ pixels from the target pixel and the target pixel is set to that mean value. With 5 variations of Gaussian blur, 5 features are generated for each image.
- Median is calculated from the pixels within a radius of σ pixels from the target pixel and the target pixel is set to that median value. With 5 variations of Gaussian blur, 5 features are generated for each image.
- Entropy 20 Within radius σ around each pixel, generates the histogram of that circle using $n = 32$, 64, 128, 256 as the number of bins and calculates the entropy as $\sum -p * log2(p)$, where p is the probability of each collection in the histogram. With 5 variations of Gaussian blurs adds 20 features for each image.

To address this comment we have applied the following modifications:

- Figure 1 on page 3 is modified and the ilastik icon is removed from the classifier's box.
- The caption of Figure 1 on page 3 is modified accordingly.

Comment #4. In Solorzano et al [2] an attempt is done to overcome this problem with the introduction of an intensity insensitive feature and the introduction of deep learning features, even a segmentation is also done.

We appreciate the reviewer for bringing this reference to our attention. The paper highlights two machine learning architectures and features that are designed to be less sensitive to intensity variations, enabling more accurate cell classification without the need for sample standardization.

This work addresses an important problem and demonstrates the efficacy of well-established machine learning models, namely fully connected neural networks and gradient boosting, for cell type identification.

However, we want to clarify that our framework primarily focuses on image preprocessing, denoising, and artifact removal. This is visualized within a red box in Figure 1 of the manuscript (please refer to the caption of Figure 1 for more details). On the other hand, the referenced paper utilizes preprocessed images for cell type identification. It is important to note that the output of our pipeline can serve as input to the methods proposed in the referenced paper. To emphasize that the referenced work utilizes preprocessed data, we quote their preprocessing steps here: ”Autofluorescence was subtracted using an image of unstained tissue exposed to corresponding HIER cycles as stained slides. Prior to cell classification, core edges as well as tissue artifacts with poor marker quality were masked away.” In our framework we pose this preprocessing as a pixel classification problem enabling the removal of various types of artifacts from the imaging data in a single pixel classification step. We illustrate an example in Fig. F10, where two artifacts (background noise and necrotic regions) are removed in a single step from the image. Additional examples are included in Figure 2 in the manuscript.

We also acknowledge that machine learning-based methods are sensitive to noise and artifacts. Effective training of deep learning models requires denoised data that is free from artifacts. Alternatively, a large number of labeled data is needed for the model to distinguish between artifacts and actual positive labels. Our main assertion is that artifact removal can be framed as a pixel classification problem. Once artifacts are removed, various downstream clustering or classification methods, such as the one introduced in the suggested paper, can be applied for cell type identification.

We find the approach presented in the referenced paper intriguing, but it is out of scope for the current publication. In our future work, we intend to investigate the performance of neural networks for cell type identification. To address this comment, we have included the referenced paper in our manuscript.

- The manuscript is modified on page 2 line 57.

Comment #5. In [3] the QuPath software is used and it combines what is done here (in a single and truly platform independent framework with a friendly user interface) by extracting features like Ilastik (plus deep learning features) and using Random Forests and also Deep learning to

do segmentation. I am aware that there is a difference between the amount of channels available in the data used in [3] and this manuscript but the platform works for both.

We would like to thank the reviewer for bringing to our attention other possible software tools such as QuPath. QuPath is a publicly available and open-source software for bioimage analysis that is widely used for tissue and cell segmentation as well as cell-type identification of multiplex imaging data. We agree with the reviewer that this versatile and user-friendly software offers a powerful set of tools useful for analyzing imaging data.

However, the focus of our manuscript is to propose a solution for image denoising and artifact removal by using pixel classification. We implemented this approach using the WEKA software tool, but it can be implemented with other software tools such as QuPath, as the reviewer has pointed out. It is worth noting that the input images used with QuPath should be preprocessed to remove any noise and artifacts. Otherwise, the accuracy of tissue and cell detection may be affected by these artifacts. One can use QuPath or any other image analysis software to address these redundant noise and artifacts.

This is especially important for mass imaging data due to its sparse and pixelated nature, which leads to a low signal-to-noise ratio. Denoising mass imaging data is more challenging than fluorescence-based images where the signal counts are several orders of magnitude higher than noise. Noise and artifacts can confound the image analysis process and generate unreliable and error-prone results. Our proposed framework preprocesses the data and generates standardized FR maps that are free from noise and artifact. The output of our framework can be fed into QuPath for single-cell segmentation and cell-type identification. We modified our manuscript to include this software as a tool that can be used to implement the process of artifact removal both in introduction and in the method section.

- The manuscript is modified on page 4 line 115.
- The manuscript is modified on page 10 line 303.

Comment #6. [2] also compares with CODEX data. Why not compare your methodology with your references 2 and 4 (Leet and Goltsev)? Comparing with InForm is not very informative unless you happen to own an Akoya device, but I do like the comparison with MAUI. I would still like to see a comparison with QuPath. In general, classical image features have been shown to not be enough to capture variety and deep learning features should be explored, how to do it is another story, it has to be chosen by the researcher, all platforms, theoretical and software

already exist, so in this aspect this manuscript doesn't bring anything new. Additionally Weka is regarded in the bioinformatics scene as a more exploratory tool but not useful for scaling to bigger experiments. Speaking of which, there is no comparison in terms of time and performance, any time I use InForm it is extremely slow, although it lets you do some parameter modifications, and parameters are also not shared or discussed. How are MAUI and InForm different/similar?

We would like to thank the reviewer for this comment. The reason we utilized Polaris data and InForm software is due to the availability of a Polaris instrument in our facility, and we frequently employ InForm for data analysis. Thus, it was compelling for us to validate our framework using the software that we commonly utilize. Consequently, we selected the Polaris dataset to include an example of fluorescence-based imaging technology. Additionally, we incorporated the referenced dataset (listed as reference 2 in our manuscript) to provide an illustration of mass-based imaging technology, as suggested by the reviewer.

For pixel classification, we employed Trainable Weka Segmentation (TWS), which is a collection of library methods that combines Fiji (ImageJ) and WEKA. Similarly, QuPath's cell detection and superpixel segmentation commands utilized ImageJ as a library for standard image processing operations. Although both TWS and QuPath utilize ImageJ for image processing, TWS utilizes the WEKA framework for classification algorithms. WEKA is a popular machine learning library written in Java. On the other hand, QuPath implemented random tree using the OpenCV library. While QuPath offers user-friendly features and the capability to analyze whole slide images, TWS provides greater versatility with various classification algorithms (including Random Forest, Decision Tree Logistic, Naive Bayes, etc.) and a wider range of imaging features. We agree with the reviewer that scaling our current implementation to bigger experiments isn't trivial. However, we provide a proof of concept in this research paper that is readily available and applicable for the datasets and imaging platforms analyzed.

Since both TWS and QuPath employ ImageJ for standard image processing operations and the classification algorithm (Random Forest) is multithreaded in both implementations, we do not anticipate significant differences in terms of execution time between the two software for the size of images we analyzed.

MAUI is a tool developed for preprocessing (denoising and artifact removal) while of InForm is a proprietary software for phenotyping flouerescenced-based imaging data.

Comment #7. When Random Forests are used, it is common to show feature importance, this

TABLE T2: Classification features from Table S1 in descending order of importance for CD20 marker from breast cancer dataset. Higher values indicate more important features.

Features	Information gain ratio score
Original image	0.3192
Gaussian_blur_2.0	0.8789
Gaussian_blur_4.0	0.8744
Entropy_2_64	0.866
Entropy_2_256	0.8628
Entropy_2_128	0.8625
Difference_of_gaussians_2.0_1.0	0.8612
Mean_2.0	0.8607
Gaussian_blur_1.0	0.8547
Difference_of_gaussians_4.0_1.0	0.8373
Mean_4.0	0.8356
Entropy_1_256	0.8328
Entropy_1_128	0.8328
Mean_1.0	0.8294
Entropy_1_64	0.8251
Entropy_4_256	0.8251
Entropy_4_128	0.8248
Sobel_filter_1.0	0.8242
Entropy_4_64	0.8201
Gaussian_blur_8.0	0.7958
Difference_of_gaussians_16.0_8.0	0.7953
Sobel_filter_2.0	0.7788
Difference_of_gaussians_8.0_1.0	0.7539
Difference_of_gaussians_16.0_4.0	0.7483
Difference_of_gaussians_4.0_2.0	0.7308
Sobel_filter_0.0	0.7235
Difference_of_gaussians_8.0_4.0	0.7045
Mean_8.0	0.7001
Entropy_8_256	0.6931
Entropy_8_128	0.6928
Entropy_8_64	0.6894
Difference_of_gaussians_16.0_2.0	0.6782
Sobel_filter_4.0	0.6776
Difference_of_gaussians_8.0_2.0	0.6709
Entropy_2_32	0.6708
Gaussian_blur_16.0	0.6663
Entropy_4_32	0.6334
Difference_of_gaussians_16.0_1.0	0.6231
Entropy_1_32	0.5938
Entropy_16_128	0.5761
Entropy_16_64	0.5726
Entropy_16_256	0.5704
Sobel_filter_8.0	0.5548
Mean_16.0	0.5532
Entropy_8_32	0.5281
Entropy_16_32	0.4512
Sobel_filter_16.0	0.3255
Median_1.0	0.3148
Median_2.0	0.295
Median_4.0	0.1997
Median_8.0	0.0309
Median_16.0	0

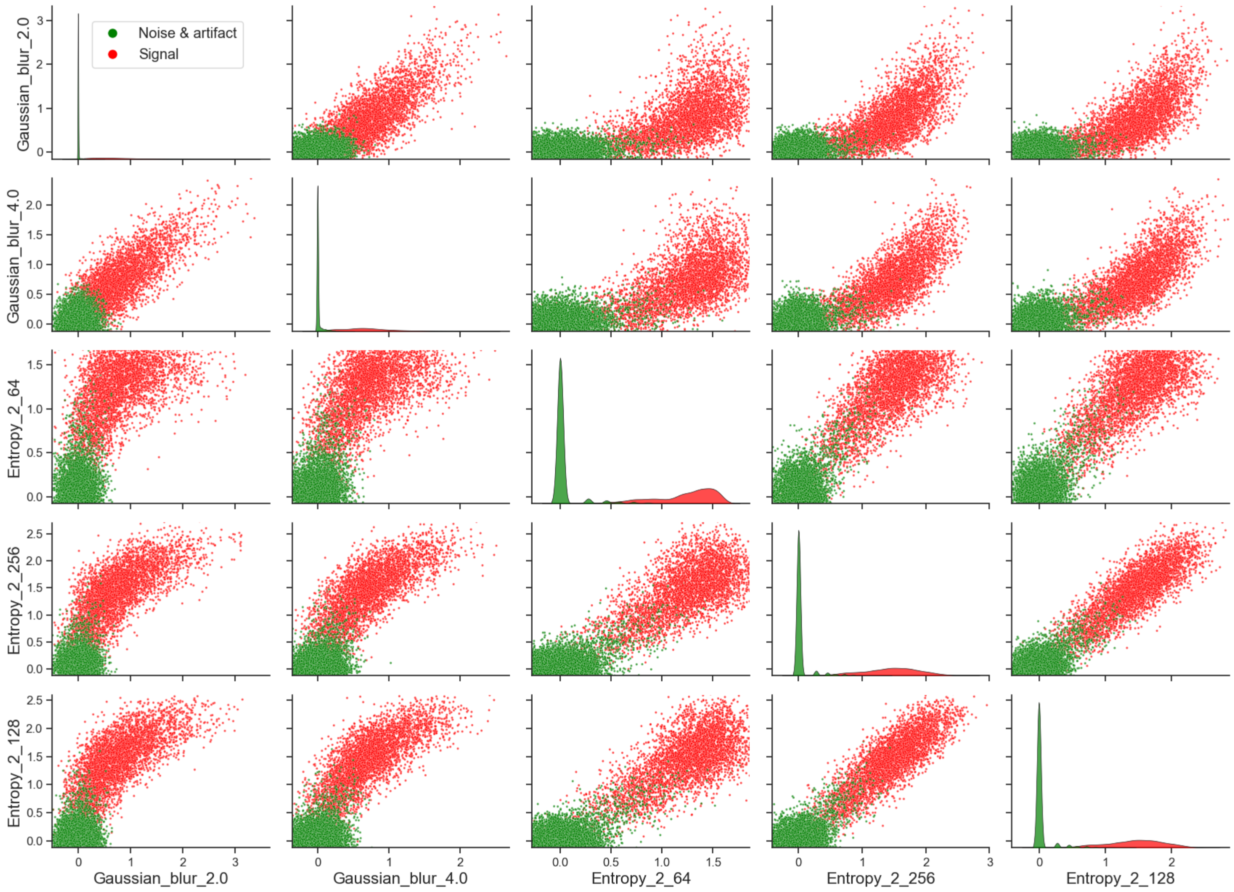

Fig. F11: The figure displays the top 5 features utilized for pixel classification of images from the CD20 channel.

way one can actually observe which features were more meaningful for the classification so this would be interesting to see. The overall idea it's still good, as a showcase of the use of simple, common and well known and used methodologies and stresses the importance of denoising and noise modeling and discusses the issue of normalization. So in summary the strength of this manuscript lies not in a novel or platform independent framework/pipeline but as a showcase of a commonly used set of methodologies and showing that it works. Data like the one used has already several studies so I am sure there are more instances to compare to.

We would like to thank the reviewer for this suggestion. To address this comment we used an arbitrarily selected marker from breast cancer dataset and computed the information gain score ratio that quantifies the importance of each feature in classification. Table T2 lists these features in descending order of importance. Furthermore, we visualized the 5 top features for the same marker in Fig. F11.

Cell types identified by our framework (rows) vs. Cell types identified by InForm (columns)

	B	CD3T	CD8T	Macrophages	Tumor	Unidentified
B	0.1	0.0	0.0	0.0	0.0	0.1
CD3T	0.0	2.0	0.1	0.0	0.0	0.5
CD8T	0.0	0.2	1.5	0.0	0.2	0.9
Macrophages	0.0	0.0	0.0	2.6	0.2	1.2
Tumor	0.0	0.0	0.0	0.0	53.6	9.4
Unidentified	0.0	0.1	0.0	0.1	2.7	24.5

Cell types identified by our framework (rows) vs. Cell types identified by TNBC[2] (columns)

	B	CD3T	CD4T	CD8T	DC	Endothelial	Macrophages	Mesenchymal	NK	Neutrophils	Tregs	Tumor	Unidentified	other immune
B	4.40	0.00	0.00	0.00	0.00	0.01	0.08	0.03	0.00	0.00	0.00	0.11	0.01	0.11
CD3T	0.00	1.52	0.15	0.00	0.00	0.00	0.11	0.00	0.00	0.00	0.00	0.00	0.00	0.16
CD4T	0.05	0.39	5.48	0.38	0.08	0.00	0.49	0.00	0.02	0.05	0.00	0.06	0.00	0.23
CD8T	0.11	0.02	0.53	7.53	0.08	0.00	0.41	0.01	0.03	0.07	0.00	0.28	0.01	0.22
DC	0.05	0.00	0.00	0.00	0.46	0.00	0.02	0.00	0.00	0.00	0.00	0.01	0.00	0.00
Endothelial	0.00	0.00	0.00	0.00	0.00	0.99	0.00	0.00	0.00	0.00	0.00	0.24	0.00	0.00
Macrophages	0.00	0.00	0.06	0.01	0.00	0.00	9.00	0.00	0.01	0.00	0.00	0.07	0.00	0.02
Mesenchymal	0.00	0.00	0.00	0.00	0.00	0.05	0.00	4.01	0.00	0.00	0.00	0.95	0.03	0.00
NK	0.00	0.00	0.00	0.00	0.00	0.00	0.00	0.02	0.27	0.00	0.00	0.03	0.00	0.00
Neutrophils	0.00	0.00	0.00	0.00	0.00	0.00	0.04	0.00	0.00	1.40	0.00	0.01	0.00	0.00
Tregs	0.00	0.00	0.01	0.01	0.00	0.00	0.01	0.00	0.00	0.00	0.67	0.01	0.00	0.00
Tumor	0.00	0.00	0.00	0.00	0.00	0.00	0.24	0.04	0.00	0.00	0.01	49.72	0.00	0.20
Unidentified	0.00	0.00	0.00	0.00	0.00	0.00	0.00	0.00	0.00	0.00	0.00	0.16	0.74	0.00
other immune	0.00	0.00	0.05	0.00	0.00	0.00	0.08	0.01	0.00	0.00	0.00	0.28	0.08	6.65

Fig. F9: **Comparison between the cell types identified in the TNBC study versus our framework.** Table entries indicate the percentage of cells in the dataset where columns and rows, respectively, compare their identified types by the baselines and our framework. The summation of the diagonal which indicates the agreement between the baseline and clustering result is about 85% for both datasets. clustering.

To address this comment we have modified our manuscript as follow:

- Table S2 is added to the supplementary text.
- Figure S3 is added to the supplementary text on page 19.

Comment #8. Figure S1 does worry me a bit as there is what seems to be a big confusion with Macrophages and Tumor, which is actually common in MIBI and mIF data. While the plot looks nice, numbers would be more appreciated and easy to compare to.

We would like to thank the reviewer for this suggestion, and we agree that presenting the information in a table format, comparing the number or percentage of cells, can provide a more informative representation compared to the plot. Another reviewer also mentioned this suggestion for Figure 2. Therefore, we have replaced both figures with tables. In these tables, the entries represent the percentage of cells in the dataset, allowing for a direct comparison of identified cell types between the baselines (columns) and our framework (rows).

Based on the summaries provided in both tables, we observe that there is reasonable agreement

between the baseline and our clustering results. To address this comment we have applied the following modifications to our manuscript:

- The scatter plot in Figure 2-b (page 8) is replace with the described matrix for the ovarian cancer dataset. The caption of Figure 2 is updated accordingly.
- The manuscript is modified on page 7, lines 220-226 to account for the change in Figure 2.
- The scatter plot in Figure S1-c is replace with the described matrix for the breast cancer dataset. The caption of Figure S1 is updated accordingly.

Comment #9. A final comment is that at the time I finished this review, the repository's readme doesn't really explain what is contained in the rest of the code and was last updated 5 months ago with no modifications (even if it says "repo under development" there has been no development. At least the code has comments which is good. Having to separate the images into channels out side the code makes it an additional step that contributes to this work not truly being platform independent, there are many steps and requirements that are not really discussed.

We appreciate the reviewer for bringing this to our attention. We have made the necessary updates to our Github repository to address the comment. The repository is now up to date, and it includes all the codes and data required for the analysis.

Specifically, we have added the scripts we used for extracting channels, organizing the data, and labeling them using ImageJ Macro scripts. These scripts are written in the same language to ensure consistency and ease of use. Furthermore, we have included the ovarian cancer dataset that we collected in-house and used in this manuscript. We note that the breast cancer dataset is publicly available [R8].

To facilitate the reproducibility of our results, we have also provided the models and (training) data generated for each (marker) for the analysis presented in the manuscript. To guide users in running the codes, we have expanded the README file. It now includes detailed instructions on how to install Fiji and WEKA, which are the required software tools for the pipeline. The README file also includes figures that serve as examples and provide a visual representation of the pipeline. With these comprehensive instructions, users will have clear guidance on how to run the codes successfully.

Thank you for your valuable feedback, and we believe that these updates address your comment appropriately.

REFERENCES

[R1] Christian Rickert, Kimbery R Jordan, "CU-IScore, https://doi.org/10.5281/zenodo.4599591," 2021.

[R2] Frency Varghese, Amirali B Bukhari, Renu Malhotra, and Abhijit De, "Ihc profiler: an open source plugin for the quantitative evaluation and automated scoring of immunohistochemistry images of human tissue samples," *PloS one*, vol. 9, no. 5, pp. e96801, 2014.

[R3] S Chevrier, HL Crowell, VRT Zanotelli, S Engler, MD Robinson, and B Bodenmiller, "Compensation of signal spillover in suspension and imaging mass cytometry. cell syst 6 (5): 612–620. e5," 2018.

[R4] Alex Baranski, Idan Milo, Shirley Greenbaum, John-Paul Oliveria, Dunja Mrdjen, Michael Angelo, and Leeat Keren, "Maui (mbi analysis user interface)—an image processing pipeline for multiplexed mass based imaging," *PLoS computational biology*, vol. 17, no. 4, pp. e1008887, 2021.

[R5] Heeva Baharlou, Nicolas P Canete, Anthony L Cunningham, Andrew N Harman, and Ellis Patrick, "Mass cytometry imaging for the study of human diseases—applications and data analysis strategies," *Frontiers in immunology*, vol. 10, pp. 2657, 2019.

[R6] Yue J Wang, Daniel Traum, Jonathan Schug, Long Gao, Chengyang Liu, Mark A Atkinson, Alvin C Powers, Michael D Feldman, Ali Naji, Kyong-Mi Chang, et al., "Multiplexed in situ imaging mass cytometry analysis of the human endocrine pancreas and immune system in type 1 diabetes," *Cell metabolism*, vol. 29, no. 3, pp. 769–783, 2019.

[R7] Nicolas Damond, Stefanie Engler, Vito RT Zanotelli, Denis Schapiro, Clive H Wasserfall, Irina Kusmartseva, Harry S Nick, Fabrizio Thorel, Pedro L Herrera, Mark A Atkinson, et al., "A map of human type 1 diabetes progression by imaging mass cytometry," *Cell metabolism*, vol. 29, no. 3, pp. 755–768, 2019.

[R8] Leeat Keren, Marc Bosse, Diana Marquez, Roshan Angoshtari, Samir Jain, Sushama Varma, Soo-Ryum Yang, Allison Kurian, David Van Valen, Robert West, et al., "A structured tumor-immune microenvironment in triple negative breast cancer revealed by multiplexed ion beam imaging," *Cell*, vol. 174, no. 6, pp. 1373–1387, 2018.
