# Supplementary material for: A platform-independent framework for phenotyping of multiplex tissue imaging data
Source: PLoS Comput Biol. 2023 Sep 21;19(9):e1011432. doi: 10.1371/journal.pcbi.1011432 (PMC10547204; doi:10.1371/journal.pcbi.1011432)
Supplement: Attachment — Submitted filename: Response_Letter_PCOMPBIOL_D_22_01763 (2).pdf [file pcbi.1011432.s004.pdf]

Responses to the Reviewers' Comments on PLOS Computational Biology with reference number PCOMPBIOL-D-22-01763

"A Platform-Independent Framework for Phenotyping of Multiplex Tissue Imaging Data"

By Mansooreh Ahmadian, Christian Rickert, Angela Minic, Julia Wrobel, Benjamin G Bitler, Fuyong Xing, Michael Angelo, Elena W.Y Hsieh, Debashis Ghosh, Kimberly R Jordan

We would like to thank the editor and the reviewers for their constructive comments, which have been helpful in the revision and improvements to the quality and technical contents of this paper. We have carefully considered and addressed all the comments and suggestions from the reviewers, as outlined in our response below. Reviewers' comments are indicated in gray italics, followed by our corresponding responses. Please note that, unless stated otherwise, all page numbers mentioned refer to the revised manuscript. In this response letter, Figures, Tables, and References are referred to as "Figure F1," "Table T1," and "Reference R1," respectively. To avoid confusion, references to figures and tables in the manuscript are made using the usual format (e.g., Figure 2, Table 3). Additionally, in the revised manuscript, all revisions are indicated using green font for easy identification.

Editor's comment

Thank you very much for submitting your manuscript "A Platform-Independent Framework for Phenotyping of Multiplex Tissue Imaging Data" for consideration at PLOS Computational Biology. As with all papers reviewed by the journal, your manuscript was reviewed by members of the editorial board and by several independent reviewers. The reviewers appreciated the attention to an important topic. Based on the reviews, we are likely to accept this manuscript for publication, providing that you modify the manuscript according to the review recommendations. Please address the comments of Reviewer 1 and add a paragraph discussing the high FR map signal/original pixel intensity relationship and how that may limit the proposed method

as suggested by Reviewer 2.

Please prepare and submit your revised manuscript within 30 days. If you anticipate any delay, please let us know the expected resubmission date by replying to this email.

When you are ready to resubmit, please upload the following:

1. *A letter containing a detailed list of your responses to all review comments, and a description of the changes you have made in the manuscript. Please note while forming your response, if your article is accepted, you may have the opportunity to make the peer review history publicly available. The record will include editor decision letters (with reviews) and your responses to reviewer comments. If eligible, we will contact you to opt in or out.*
2. *Two versions of the revised manuscript: one with either highlights or tracked changes denoting where the text has been changed; the other a clean version (uploaded as the manuscript file).*

Response to the Editor

We would like to thank the editor for handling the review process of our manuscript. We have thoroughly addressed each of the reviewers' comments and incorporated their valuable suggestions. We would like to provide you with a summary of the changes made in the revised manuscript. For a more comprehensive understanding, we kindly request that you refer to our response to the reviewers.

- In response to reviewer 1's minor comments, we made adjustments to Figure 2a, the caption of Figure 3, and Figure S1. Additionally, we cited the relevant reference suggested by the reviewer.
- To address the feedback from reviewer 2, we have integrated all three proposed options, introduced Figure S2 in the Supplementary text, and made necessary modifications to our manuscript on page 7.

Reviewer #1

I thank the authors for the revision and feel that my comments have been sufficiently addressed. Two small comments remain which should be easy to address and (minor comment#1) the authors should also cite the meanwhile not only on BioRxiv available paper from the Thorek lab (https://doi.org/10.1038/s41467-023-37123-6). (minor comment#2) Regarding major comment 2. Fig. 2A makes for better visual impression of cross-talk albeit unfortunately now the histogram for that one single CD20 positive cells is hard to appreciate. Potentially a zoom in could help. Additionally I was not aware of the dual labelling process for noise first and then artefacts. Might be worth to specify this in the methods if the authors believe that this is superior over a one step labelling (noise+artefacts). Not fully clear to me what the difference would be. (minor comment#3) Regarding minor comment 1: unclear what the difference between Fig. 3b right side and Fig. S1 is. The heatmaps look pretty much identical.

We would like to thank the reviewer for these valuable comments. To ensure that we have addressed this comment appropriately, we have added comment numbers to the text. We did not alter the order or content of the comments, but rather added comment numbers as a means of ensuring that all concerns have been adequately addressed.

Minor comment#1: *the authors should also cite the meanwhile not only on BioRxiv available paper from the Thorek lab (https://doi.org/10.1038/s41467-023-37123-6).*

We would like to thank the reviewer for bringing this publication to our attention. We have cited this paper in the introduction section where we are introducing various available denoising and artifact removal frameworks/pipelines.

Minor comment#2: *Regarding major comment 2. Fig. 2A makes for better visual impression of cross-talk albeit unfortunately now the histogram for that one single CD20 positive cells is hard to appreciate. Potentially a zoom in could help. Additionally I was not aware of the dual labelling process for noise first and then artefacts. Might be worth to specify this in the methods if the authors believe that this is superior over a one step labelling (noise+artefacts). Not fully clear to me what the difference would be.*

Following the reviewer's suggestion we have regenerated the histograms in Figure 2a for an inset of the displayed image.

Regarding the concurrent or subsequent annotation of noise and artifacts, we would like to

clarify that during our training process, we treat the signal as the positive class (class I) and consider all other artifacts and noise as the negative class (class II). These classes are annotated together in a single step. The intermediate figures presented in the previous response letter were provided solely to clarify that each artifact exhibits distinct characteristics, necessitating the inclusion of examples of those artifacts in the training sets. While it is possible to annotate noise and artifacts separately in multiple steps, we did not adopt this approach to generate the results presented in this manuscript. We have made appropriate modifications to the manuscript to explicitly explain that our annotation of noise and different types of artifacts is conducted in a single step.

Minor comment#3: *Regarding minor comment 1: unclear what the difference between Fig. 3b right side and Fig. S1 is. The heatmaps look pretty much identical.*

We appreciate the reviewer's observation and would like to provide further clarification on the purpose of Figure 3b and Fig. S1 in our manuscript. Both figures illustrate the population of different cell types on the y-axis and the level of expression for specific markers on the x-axis, represented by the color of the heatmap.

In Figure 3b, the cells are sorted based on the cell types identified by the TNBC paper [R1] (the baseline). Conversely, in Fig. S1, the cells are sorted according to the clustering results obtained from our analysis. As the reviewer suggests, we acknowledge that there is no significant difference between these two heatmaps, indicating a strong agreement between our results and the baseline we are comparing to. We have made modifications to the manuscript to clarify the differences between these heatmaps.

To address this comment, the following modifications have been applied to the manuscript:

- Reference [R2] is cited in the manuscript on page 2, line 57, and is listed as reference# 23.
- The histograms of the displayed images in Figure 2a have been replaced with histograms of insets of those images, enhancing the demonstration. The caption of Figure 2 has been updated to reflect this change.
- The manuscript has been modified on pages 10-11, lines 523-525 to explicitly explain that our annotation of noise and different types of artifacts is conducted in a single step.
- The caption of Figure 3 and S1 has been revised to provide a clearer distinction between the heatmaps in Figures 3 and S1.

Reviewer #2

Major issue 1 - partially addressed. I thank the authors for their replies and clarifications on this issue. Firstly the addition of spearman's rank is a helpful addition. Secondly, my concerns with Figure 2d have been successfully addressed. Thirdly, including further discussion about non-linearity has been helpful. I would like to clarify my point since it may not have been clear in my original comment and some of my concern remains.

I remain concerned about the correlation of the data within the upper values of the FR map, which I believe are the most relevant values for most applications.

Many downstream analyses will focus on quantifying signal that is found at higher values of FR map (this is also higher values of original signal intensity in general). One example of how this could happen is by choosing to analyze data that is only within cells to compare cells to each other. An example of this in the paper is where it says in the Figure 1 caption "Marker expression within the border of each cell is then measured from the class I FR maps." By doing an analysis like this, the lower values of FR map become irrelevant and the overall monotonic increase is less relevant. In this scenario, the most relevant question is: is the FR map signal within the upper values monotonically increasing with original pixel intensity?

While the authors have very helpfully described this monotonic increase for the whole dataset and helpfully quantified the correlation over the whole thing, it is still a matter of concern about how well the FR map values reflect the original pixel intensity for areas of high FR map that are likely to be analyzed as part of signal. Part of my motivation for considering this is that in my mind, expression of signal certainty is not quite the same as increased signal.

This paper shows this approach works overall. However, I am trying to make this limitation, if it exists, more clear. There are a few options I can think of to resolve this problem.

- *Option 1: Add a written caveat (perhaps in the discussion) that monotonic increases are less clear for high FR values. (or something like you might lose dynamic range up at those values)*
- *Option 2: Add a supplementary figure that replicates figure 3a (row 3) but plotted on a linear scale, not on a log scale, for FR map values only above 0.5 (spearman's rank optional), just to make this area of the graph clearer (it may be that the current graph is clear enough in the editor's opinion)*
- *Option 3: A supplementary table of spearman's rank for FR map ¿0.5 for the data is figure*

3 row 3

It may be that the editor thinks there is already enough information about correlation at high FR values in the manuscript. In which case, that is fine.

Minor issue 1 - fully addressed

- *Minor issue 2 - fully addressed*
- *Minor issue 3 - fully addressed*
- *Minor issue 4 - fully addressed*
- *Minor issue 5 - fully addressed*
- *Other points 1 - fully addressed*
- *Other points 2 - fully addressed*

We would like to thank the reviewer for further clarification on this comment and providing us with three options to address the comment. We have incorporated the three proposed options to address the reviewer's comment.

Before discussing our results, it is essential to underscore an important point that enables us to provide a more comprehensive response to the reviewer's question: "*Is the FR map signal within the upper values monotonically increasing with original pixel intensity?*" To address this query, the reviewer has recommended calculating the Spearman's rank correlation coefficient for high values of the FR maps, proposing a threshold value of 0.5. However, we must consider that the high expression values or positive values that are relevant to downstream analysis may not necessarily exceed 0.5, according to our observations. Although a threshold of 0.5 may be appropriate in numerous binary classification scenarios, it does not necessarily apply to our specific case.

To illustrate this aspect, we present histograms in Figure F1, displaying average values per cell measured on FR maps for various markers. Two key observations emerge from this representation: First, the threshold value separating high FR map values relevant for identifying cell types in downstream analysis (positive cells for a given marker) from low values varies depending on the marker. Second, the threshold frequently falls below the suggested value (0.5). For instance, markers like FoxP3 and p53 exhibit thresholds as low as 0.1 and 0.15, approximately 0.2 for Vimentin, and possibly as high as 0.5 for CD209, as proposed by the reviewer. Therefore, calculating the Spearman's rank correlation coefficient for high FR map values may not necessarily align with values greater than 0.5.

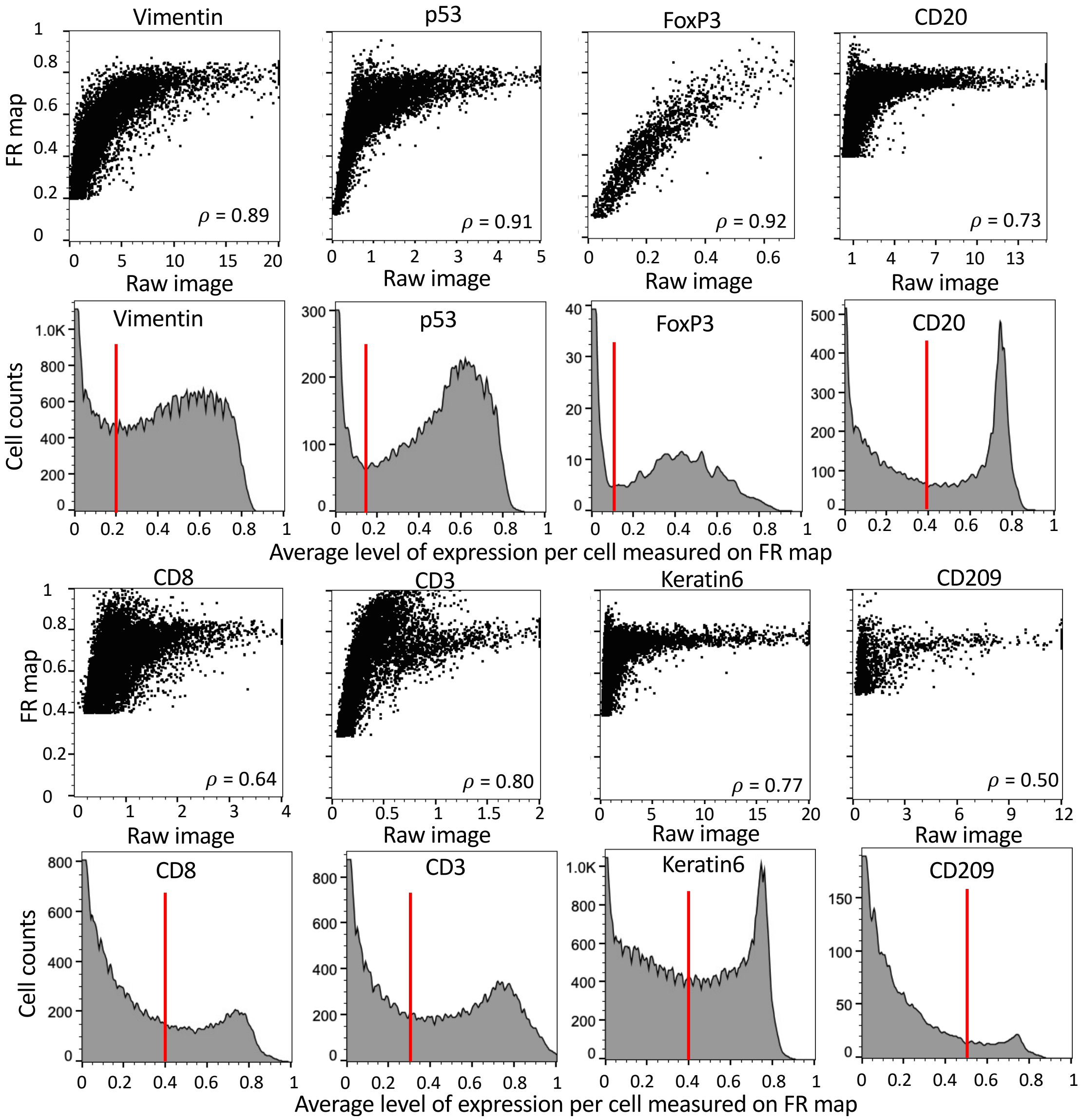

Fig. F1: **Quantifying the correlation between the raw image pixel intensity and FR maps for marker-positive cells.** Correlation plots illustrating marker expression per cell from raw images (x-axis) against FR maps (y-axis) are presented in rows 1 and 3. Specifically, these plots focus on cells where the average FR map values per cell exceed a selected threshold value. To rationalize the chosen threshold values (indicated by red lines), histograms displaying average values per cell measured from FR maps are included, delineating positive cells for a given marker from the negative ones.

To address this concern, we created correlation plots between marker expression per cell from raw images (x-axis) and FR maps (y-axis) in Fig. F1, employing a linear scale for cells where the FR map values exceeded a specific threshold. To justify the chosen threshold, we included histograms representing average values per cell measured on FR maps, which exhibit a bi-

modal distribution, facilitating the identification of a more appropriate threshold. Furthermore, we quantified the correlation by calculating the Spearman's rank correlation coefficient.

Finally, we would like to emphasize a few crucial points to address the reviewer's comment: We acknowledge that the mapping from pixel values in the raw image to the FR map is influenced not only by pixel intensity but also by the spatial information of surrounding pixels (as depicted in Figure 2c of the manuscript). Consequently, positive signals may yield large values in the FR map; however, as these values increase in the raw image, the values level off in the FR map. This characteristic does not present any issues, as our framework is not designed to assess the level of expression for functional markers, but rather to determine whether a cell is positive or negative for a given marker.

To address this comment, we have applied the following modifications to our manuscript:

- Figure F1 is included in the Supplementary text as Figure S2.
- Them manuscript is modified on page 7 lines 212-214 to refer to the supplementary text.

References

[R1] Leeat Keren, Marc Bosse, Diana Marquez, Roshan Angoshtari, Samir Jain, Sushama Varma, Soo-Ryum Yang, Allison Kurian, David Van Valen, Robert West, et al., "A structured tumor-immune microenvironment in triple negative breast cancer revealed by multiplexed ion beam imaging," *Cell*, vol. 174, no. 6, pp. 1373–1387, 2018.

[R2] Peng Lu, Karolyn A Oetjen, Diane E Bender, Marianna B Ruzinova, Daniel AC Fisher, Kevin G Shim, Russell K Pachynski, W Nathaniel Brennen, Stephen T Oh, Daniel C Link, et al., "IMC-Denoise: a content aware denoising pipeline to enhance imaging mass cytometry," *Nature communications*, vol. 14, no. 1, pp. 1601, 2023.
