# Supplementary material for: A platform-independent framework for phenotyping of multiplex tissue imaging data
Source: PLoS Comput Biol. 2023 Sep 21;19(9):e1011432. doi: 10.1371/journal.pcbi.1011432 (PMC10547204; doi:10.1371/journal.pcbi.1011432)
Supplement: Attachment — Submitted filename: Review.docx [file pcbi.1011432.s002.docx]

Review

The authors touch upon an interesting and timely topic which deals with different aspects of preprocessing of raw data from multiplexed imaging. They argue that downstream analysis of imaging data relies on artefact removal, denoising and normalization which could all affect cell type identification and sample-to-sample quantification. The propose a single-step cross platform solution which relies on pixel classification which includes denoising, artefact removal and normalization and does not rely on any user defined thresholds.

In this method each pixel from each channel is classified as either signal or noise and this is outputted as probability which ranges from 0 to 1 (FR map) and the authors consider these values as normalized and use them for downstream analysis.

While the manuscript is compact, easy to read and interesting there are some aspects that are unclear to me and need clarification.

Major comments:

- It is unclear how the training and the validation of the approach were done. Did the authors train on the entire stack of the individual images? If so, then no validation of the approach is provided. Meaning, how does the classifier perform if it is provided data from a sample/patient it has never seen? Usually the specificity and sensitivity of random forests drops then. The authors should make this clear and if the whole stack was used for training then the authors should rerun the classifier with a hold out test set which they can then use for testing. This has implications for the applicability of the approach as it would likely not be feasible to label 40 channels of projects that contain e.g. 400 images… Thus, knowing the performance on new data is crucial.
- Fig 2:
  - With respect to the general commen above, it is unclear for all panels what the training was done on. Where these images trained individually, were they part of a training set or were they part of a test set?
  - In panel A it does not seem that 163 shows cross-talk in 164, meaning the pattern of 163 cannot be seen in 164. It is also unclear what exactly the purple arrows point at (similarly for panels B and C). Why do the authors believe that vimentin expression can be observed in the SMA channel?
  - In panel D, again, the authors should clarify the process of how the FR map was generated for this specific image. If the density of pixels is taken into account, then I would still expect to see the signal from cell C propagate to the FR map. However in the FR map there is no visible signal any more. The authors should clarify this.
- The autors show in Fig. 3a (and 4a) the correlation between the original raw values and the FR map values. Please provide rank based correlation coefficients. The question is, if the FR map, which is a mere probability of a pixel classified as signal or noise, is correlated with the original raw values. If not one needs to be carefuly when comparing expression levels for functional markers such as Ki67. Please also provide the corellation for Ki67. If no high correlation is given (> 0.8) I would suggest that the authors should clearly state this limitation in the manuscript and point to the validity of phenotyping but limitations regarding expression level comparisons.
- Please provide statistics throughout the manuscript when arguing for significance.
- There seem to be duplicated references. E.g. 21 and 27, or 19 and 26. Please correct.

Minor comments

- The authors should provide single cell heatmaps depicting the FR map expression of the detected cell types for both datasets to see the result of the clustering. Displaying a sub-sample of the data should be fine (e.g. 10000 cells).
- Fig. 3c: unclear why “predicted” cell types? Are these not simply the cell counts based on phenotyping?
- In Fig. S1C and Fig. 4b the authors should provide a better display of the data for comparison. The values of the confusion matrix should be shown instead of the black dots.
- In the discussion the authors state that the approach requires less hands on time but is more computationally intense. However, it is unclear to me if manually labelling 40 channels can be considered low manual input. If I calculate correctly, then labelling 40 channels takes 40*10 min ~ 5 hours. On top there will be roughly 4000 mins (~3 days) of FR map calculation for a fairly small dataset of 41 images. With respect to expert knowledge, do the authors believe that labelling signal and noise for 40 channels requires less knowledge than thresholding or other manual curation steps? So please specify if by “high runtime” you only mean computational or also hands on time and clarify in the text and potentially indicate that labelling images also requires some expert knowledge.
- The authors should add this paper to introduction and potentially discuss differences and advantageous of their approach. PMID 34196108
